# Multicomponent Nitride/High-Entropy Alloy Heterostructures as Highly Active and Stable Electrocatalysts for Ampere-Level Water Oxidation

**DOI:** 10.1007/s40820-026-02316-3

**Published:** 2026-09-11

**Authors:** Xin-Ying Sun, Tian-Yi Dai, Rui-Qi Yao, Yun-Fei Chang, Zhi-Lan Zhou, Ying Wang, Tong-Hui Wang, Gao-Feng Han, Zi Wen, Hang Shi, Xing-You Lang, Qing Jiang

**Affiliations:** 1 Key Laboratory of Automobile Materials (Jilin University), Ministry of Education, School of Materials Science and Engineering, Jilin University, Changchun, 130022 People’s Republic of China; 2 Key Laboratory of Polyoxometalate and Reticular Material Chemistry of Ministry of Education, Faculty of Chemistry, Northeast Normal University, Changchun, 130024 People’s Republic of China

**Keywords:** Multicomponent nitride, High-entropy alloy, Nanoporous metals, Electrocatalysts, Oxygen evolution reaction

## Abstract

Developing highly active and stable oxygen evolution reaction catalysts is paramount for cost-effective large-scale green hydrogen production via water electrolysis. Here we report nonprecious metal-based multicomponent nitride/high-entropy alloy heterostructures as cost-effective electrocatalysts to efficiently mediate alkaline water oxidation by in-situ configurating cooperative Brønsted basic oxyanion-solid interfaces during phase transformation of multicomponent nitride into amorphous N-doped NiFeCoMoCrOOH along with partial dissolution of high-value metals of Mo and Cr to form molecular MoO_4_^2−^ and CrO_4_^2−^ species. The protonation of Brønsted basic oxyanions facilitates the critical *OOH intermediate formation on N-doped NiFeCoMoCrOOH with near-optimal adsorption energy, substantially accelerating the oxygen evolution reaction kinetics. Consequently, the heterostructure electrode exhibits superior oxygen evolution reaction electrocatalysis in 1 M KOH solution, delivering ultrahigh current density of ~ 2.5 A cm^−2^ at low overpotential of 300 mV. Its alkaline water electrolyzer operates stably at ampere-level current density for > 1700 h. Our findings reveal an unconventional molecule-assisted multi-site mechanism of multicomponent catalysts to accelerate multiple-intermediate reaction.

**Supplementary Information:**

The online version contains supplementary material available at https://doi.org/10.1007/s40820-026-02316-3.

## Highlights

- Nonprecious metal-based multicomponent nitride anchored on nanoporous high-entropy alloy skeleton is constructed for ampere-level water oxidation via in-situ configurating cooperative Brønsted basic oxyanion-solid multiple active interfaces.
- The self-supported nanoporous (NiFeCo)_3_(MoCr)_3_N/HEA electrodes exhibit exceptional catalytic activity and durability by virtue of MoO_4_^2−^ and CrO_4_^2−^ ions acting as molecular co-catalysts to mediate proton spillover to facilitate the formation of *OOH.
- The alkaline water electrolyzer assembled with nanoporous (NiFeCo)_3_(MoCr)_3_N/HEA anode and (MoCr)(NiFeCo)_4_/HEA cathode can operate stably at ampere-level current density for over 1700 h.

**Supplementary Information:**

The online version contains supplementary material available at https://doi.org/10.1007/s40820-026-02316-3.

## Graphical abstract

**Figure:**
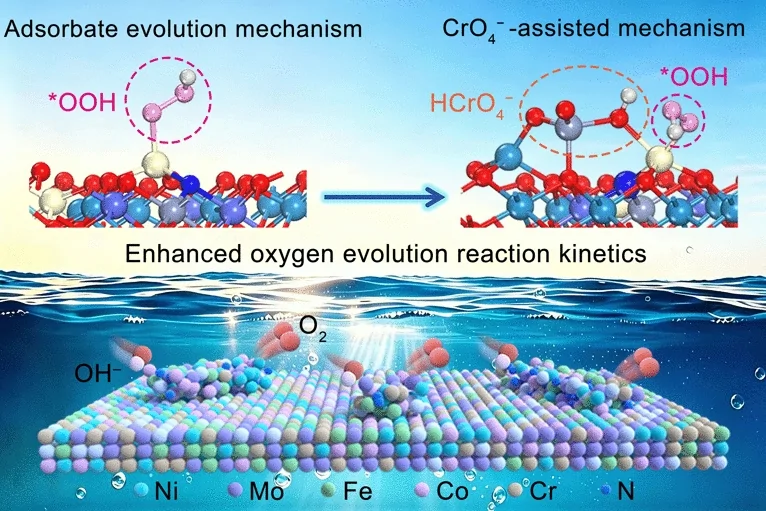

## Introduction

Water electrolysis driven by renewable electricity from solar and wind resources promises a sustainable route to carbon neutralization by making use of molecular hydrogen as a clean and high-density alternative to fossil fuels for meeting future energy needs [1–4]. However, it often undergoes insufficient operating efficiency no matter in mature alkaline water electrolyzer (AWE) or emerging anion exchange membrane water electrolyzer [4–6], leading to the present dilemma that water electrolysis only accounts for ~ 4% of global hydrogen production [3, 5, 7, 8]. This is primarily due to sluggish kinetics of anodic oxygen evolution reaction (OER), also known as water oxidation, which involves multiple oxygen intermediates (*OH, *O, *OOH) with multi-proton-coupled electron transfer (PCET) processes [7, 9, 10]. Even mediated by IrO_2_ or RuO_2_ as the benchmark OER catalyst [9, 11, 12], it takes substantial overpotentials to reach industrial-scale current densities because of universal linear scaling relationships (LSRs) that limit the reaction kinetics as a consequence of incongruous adsorption/desorption of multiple oxygen intermediates on such conventional single-site catalysts [10, 13, 14]. Therefore, developing unconventional and inexpensive electrocatalysts with efficient and robust OER performance at ampere-scale current densities is crucial to promoting widespread use of water electrolysis technologies for large-scale green hydrogen production. So far, considerable strides have been made in the development of bimetallic or multi-metallic catalysts based on first-row (3*d*) late transition metals (for example, Fe, Co and Ni), including their oxides/hydroxides [9, 15, 16], sulfides [17, 18], phosphides [19, 20] and molecular complexes with incorporation of additional metallic or/and non-metallic elements [10, 21, 22]. These materials mediate the alkaline OER in either the adsorbate evolution mechanism (AEM) [23–25] or the lattice oxygen mechanism (LOM) [26–28] with remarkably enhanced intrinsic activity by optimizing adsorption energies or breaking the LSRs to accelerate reaction kinetics. Nevertheless, most bimetallic or multi-metallic compounds encounter severe activity degradation due to selective dissolution of metal component or surface structure collapse under the harsh electrooxidation environments [15, 22, 29, 30], far from satisfying the long-lifetime requirement (> 1000 h) of water electrolysis [9, 10, 31]. Accordingly, it is of high desire to explore novel materials with high activity and stability as efficient OER electrocatalysts.

Here we report self-supported nanoporous multicomponent nitride/alloy heterostructure made of Fe, Co, Ni late transition metals and Cr, Mo high-valence metals in (NiFeCo)_3_(MoCr)_3_N complex nitride and NiFeCoMoCr high-entropy alloy [(NiFeCo)_3_(MoCr)_3_N/HEA] for efficient alkaline OER electrocatalysis via in-situ configuring cooperative Brønsted basic oxyanion-solid multiple active interfaces. Owing to the thermodynamically less stability of (NiFeCo)_3_(MoCr)_3_N complex nitride, it undergoes phase transformation into amorphous N-doped NiFeCoMoCr oxyhydroxide grafted on HEA skeleton (NiFeCoMoCrOOH-N/HEA), along with partial dissolution of Mo and Cr atoms to generate MoO_4_^2−^ and CrO_4_^2−^ Brønsted basic oxyanions, under water oxidation conditions. These Brønsted basic oxyanions electrostatically accumulate around the NiFeCoMoCrOOH-N surface to form cooperative solid-molecular interfaces, where the MoO_4_^2−^ and CrO_4_^2−^ ions act as molecular co-catalysts to mediate proton spillover from *OH intermediate adsorbed on Fe top site, facilitating the critical O-O bond formation in *OOH intermediate with a low energy barrier to boost reaction kinetics. Furthermore, the solid-molecular multiple active interfaces effectively depress overoxidation of metal components, improving the electrochemical durability of solid NiFeCoMoCrOOH-N phase. Associated with HEA skeleton that endows sufficient active sites and accelerates electron transfer and ion/molecule transportation, nanoporous (NiFeCo)_3_(MoCr)_3_N/HEA exhibits exceptional OER electrocatalysis to deliver ultrahigh current density of ~ 2.5 A cm^−2^ at low overpotential of 300 mV. The AWE assembled with nanoporous (NiFeCo)_3_(MoCr)_3_N/HEA as the anode and nanoporous HEA supported complex intermetallic (MoCr)(NiFeCo)_4_ [(MoCr)(NiFeCo)_4_/HEA] as the cathode, i.e., (NiFeCo)_3_(MoCr)_3_N/HEA||(MoCr)(NiFeCo)_4_/HEA, can achieve ~ 1.25 A cm^−2^ at a low cell voltage of 1.92 V without iR compensation and maintain outstanding stability for over 1700 h.

## Experimental Section

### Preparation of Nanoporous Electrodes

Self-supported nanoporous multicomponent nitride/alloy heterostructure electrodes were fabricated by facile and scalable alloying/dealloying technique, followed by annealing procedures in air and then in Ar/NH_3_ mixture atmosphere. Precursor alloy ingots of Ni_25–*w*–*x*–*y*–*z*_Fe_*w*_Co_*x*_Mo_*y*_Cr_*z*_Al_75_ (*w*, *x*, *z* = 0 or 2, *y* = 0 or 6, at%) were first produced by arc melting pure Ni and Al metals with/without the addition of Fe, Co, Mo or/and Cr under Ar atmosphere. These alloys ingots were sliced into sheets with thickness of ~ 400 µm and then chemically dealloyed to prepare nanoporous electrodes in N_2_-saturated 6 M KOH solution at 70 °C until there are no bubbles to generate. After rinsing in ultrapure water to clean residual chemicals and then drying in a vacuum environment, the as-prepared nanoporous multicomponent intermetallic compound/high-entropy alloy hybrid materials were further annealed in air atmosphere at 300 °C for 2 h and then in a mixed atmosphere of Ar/NH_3_ with a molar ratio of 90:10 at 900 °C for 2 h to prepare multicomponent nitride/alloy heterostructure electrodes. For comparison, nanoporous multicomponent oxide/alloy electrodes were prepared by annealing at 300 °C for 2 h in air atmosphere and at 900 °C for 2 h in Ar atmosphere. In addition, RuO_2_ and Pt/C inks were fabricated by uniformly mixing commercially available RuO_2_ (99.9%, Sigma Aldrich) and Pt/C (20 wt%, Johnson Matthey) into isopropanol aqueous solution containing proper Nafion (0.05 wt%, Sigma Aldrich) by rigorous sonication, and then dropping onto nanoporous Ni with the mass loading of 2 mg cm^−2^ for electrochemical measurement.

### Physicochemical Characterizations

Microstructure and chemical composition of precursor alloys, multicomponent nitride/alloy heterostructure electrodes were conducted on a field-emission scanning electron microscopy (SEM, JSM-7900F, 5 kV) equipped with X-ray energy-dispersive spectroscopy (EDS) and a field-emission transmission electron microscopy (TEM, JEOL JEM-2100F, 200 kV). X-ray diffraction (XRD) measurements were carried out on a Rigaku Smartlab diffractometer at a power of 9 kW with a monochromated Cu *K*_α_ radiation. Surface chemical states of electrocatalysts were characterized by X-ray photoelectron spectroscopy (XPS, Thermo ECSALAB 250) equipped with an Al anode, where charging effects were compensated based on the C 1*s* peak with the binding energy of 284.8 eV. The Ni, Fe, Co, Mo, and Cr K-edge X-ray absorption fine structure spectra were recorded by using the easyXAFS 300 system (easyXAFS, US). The metal ion concentrations in electrolyte were detected by inductively coupled plasma optical emission spectroscopy (ICP-OES, Thermo electron). Raman spectra were performed on a micro-Raman spectrometer (Renishaw) equipped with a 532-nm-wavelength laser at a power of 1 mW.

### Electrochemical Measurements

All OER electrocatalytic characterizations were conducted in a classic three-electrode setup, where self-supported nanoporous electrodes were directly used as the working electrodes, a carbon rod as the counter electrode and an Ag/AgCl electrode as the reference electrode, 1 M KOH as the electrolyte. To assess the OER performance of nanoporous electrodes, linear scanning voltammetry (LSV) was conducted with 100% iR- compensation at a scan rate of 1 mV s^−1^ at 25 °C (i was the real working current and R is the measured series resistance). Electrochemical impedance spectra (EIS) were collected at the overpotential of 100 mV in the frequency range from 10 mHz to 100 kHz with 5 mV amplitude. In order to evaluate the electrochemical surface areas (ECSAs) of these electrodes, cyclic voltammetry (CV) curves were obtained in the non-faradaic potential window of − 0.1 to 0 V (versus Ag/AgCl) at scan rates of 20, 25, 30, and 35 mV s^−1^. The OER durability measurements of nanoporous (NiFeCo)_3_(MoCr)_3_N/HEA, Ni_3_Mo_3_N/Ni and (NiFeCo)_2_(MoCr)_3_O_8_/HEA electrodes were performed at the overpotential of 270 mV for 1600, 450 and 200 h, respectively. (NiFeCo)_3_(MoCr)_3_N/HEA||(MoCr)(NiFeCo)_4_/HEA AWE was constructed by assembling (NiFeCo)_3_(MoCr)_3_N/HEA as the anode and (MoCr)(NiFeCo)_4_/HEA as the cathode in 1 M KOH electrolyte for investigating the overall water splitting performance. The polarization curves of the electrolyzers were collected at a scan rate of 1 mV s^−1^ at 25 °C and the stability was tested at the cell voltage of 1.92 V for > 1700 h.

### Theory Computations

All the spin-polarized density functional theory (DFT) calculations were performed based on the Vienna ab initio simulation package (VASP) code with Hubbard-*U* corrections, and the projector-augmented wave (PAW) method was employed. The Perdew-Burke-Ernzerhof (PBE) of generalized gradient approximation (GGA) functional was used to describe electronic exchange-correlation interactions. The Grimme method (PBE-D3) correction was adopted to include the van der Waals interactions. The effective Hubbard-*U* parameters (*U*-*J* values) were set to be 5.00 eV for Cr, 4.30 eV for Fe, 3.32 eV for Co, 5.50 eV for Ni and 4.38 eV for Mo. The 4 × 4 × 2 supercell of γ-NiOOH (001) surface was established with a vacuum in the *z* direction of 15 Å, and then Ni atoms were randomly replaced by Fe, Co, Mo and Cr atoms to simulate the NiFeCoMoCrOOH structure, where the atomic ratios were taken from experimental results. To build the amorphous structure, ab initio molecular dynamics (AIMD) calculation under the NVT condition was performed at 900 K for 10 ps with a time step of 1 fs. Another AIMD calculation was conducted at 300 K for 5 ps, followed by a geometry optimization calculation to stabilize the amorphous configuration. The cutoff energy was set to be 400 eV, and the *k*-point grids of Brillouin zone were set to 3 × 3 × 1. The convergence criteria for energy and force were set to be 1.0 × 10^−5^ eV and 0.02 eV Å^−1^, respectively. The binding energy (∆*E*_b_) was determined according to the equation: ∆*E*_b_ = E_*M_–E_*_–*E*_M_, where E_*M_ represents the total energy of adsorption system, E_*_ and *E*_M_ correspond to the isolated energy of pristine catalyst and adsorbate, respectively. Besides, the reaction Gibbs free energy (∆*G*) was calculated according to the equation ∆*G* = ∆*E* + ∆ZPE − *T*∆*S*, where ∆*E*, ∆ZPE and ∆*S* represented the change of total energy, zero-point energy and entropy, respectively.

## Results and Discussion

### Preparation and Physicochemical Characterizations

**Fig. 1:**
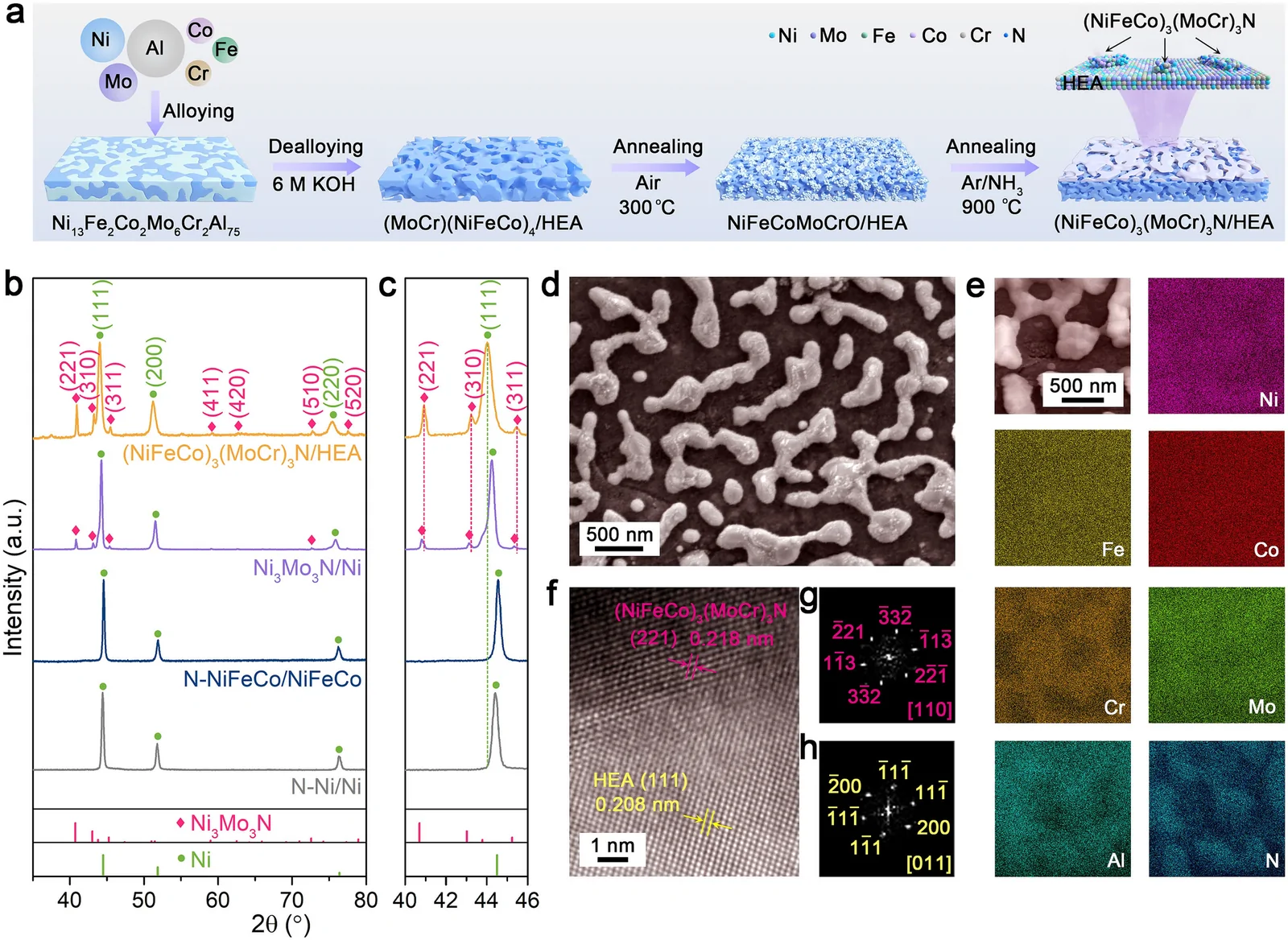
Preparation and microstructure characterization of nanoporous multicomponent electrocatalysts. **a** Schematic illustration for fabrication of nanoporous (NiFeCo)_3_(MoCr)_3_N/HEA heterostructure electrode by facile alloying/dealloying technique, followed by thermal oxidation and nitridation procedures. **b** XRD patterns of nanoporous (NiFeCo)_3_(MoCr)_3_N/HEA, Ni_3_Mo_3_N/Ni, N-NiFeCo/NiFeCo and N-Ni/Ni electrode. The line patterns show reference cards 49-1336 for orthorhombic Ni_3_Mo_3_N and 04-0850 for face-centered cubic Ni according to JCPDS. **c** Diffraction patterns in the range of 2θ from 40° to 46° for nanoporous (NiFeCo)_3_(MoCr)_3_N/HEA, Ni_3_Mo_3_N/Ni, N-NiFeCo/NiFeCo and N-Ni/Ni electrodes. **d** Representative SEM image of nanoporous (NiFeCo)_3_(MoCr)_3_N/HEA electrode, displaying (NiFeCo)_3_(MoCr)_3_N nanoislands with a width of ~ 200 nm are seamlessly integrated on HEA skeleton. **e** SEM image of nanoporous (NiFeCo)_3_(MoCr)_3_N/HEA heterostructure and the corresponding EDS elemental mappings for Ni, Fe, Co, Cr, Mo, Al and N. **f** Typical HRTEM image of (NiFeCo)_3_(MoCr)_3_N/HEA heterostructure interface. **g**, **h** FFT patterns of (NiFeCo)_3_(MoCr)_3_N (**g**) and HEA (**h**) region in **f**

The self-supported nanoporous (NiFeCo)_3_(MoCr)_3_N/HEA heterostructure electrode is fabricated by a facile alloying/dealloying technique [32–34], followed by annealing procedures in air and subsequently in Ar/NH_3_ mixture atmosphere (Fig. 1a). Briefly, multicomponent Ni_13_Fe_2_Co_2_Mo_6_Cr_2_Al_75_ alloy is first prepared by arc-melting pure Al, Ni, Fe, Co, Mo, and Cr metals in atomic ratio (at%) of 75: 13: 2: 2: 6: 2 (Fig. S1) and then solidifying to form a multiphase microstructure composed of immiscible α-Al [32, 35], intermetallic Al_3_Ni [36, 37], Al_13_Fe_4_ [35, 38], Al_9_Co_2_ [36, 39], Al_4_Mo [35, 39] and Al_84.6_Cr_15.4_ [38], in addition to nanoscale precipitations of chemically complex intermetallic (MoCr)(NiFeCo)_4_ [39] (Fig. S2). During the chemical dealloying in N_2_-pured 6 M KOH solution, selectively etching α-Al phase and less-noble Al component of intermetallic compounds gives rise to the formation of nanoporous (MoCr)(NiFeCo)_4_/HEA [36, 39], where the complex intermetallic (MoCr)(NiFeCo)_4_ nanoprecipitations are in-situ anchored on NiFeCoMoCrAl HEA ligaments (Fig. S3). When annealing in air at 300 °C and subsequently in Ar or Ar/NH_3_ mixture atmosphere at 900 °C, there takes place thermal oxidation or nitridation to generate (NiFeCo)_2_(MoCr)_3_O_8_ or (NiFeCo)_3_(MoCr)_3_N on nanoporous HEA skeleton for the production of nanoporous (NiFeCo)_2_(MoCr)_3_O_8_/HEA (Fig. S4) or (NiFeCo)_3_(MoCr)_3_N/HEA, respectively. XPS survey analysis of nanoporous (NiFeCo)_3_(MoCr)_3_N/HEA attests the presence of N (Fig. S5), in addition to Ni, Fe, Co, Mo, Cr, Al with atomic ratio of 59: 9: 9: 10: 4: 9 determined by EDS (Fig. S6a). XRD patterns demonstrate the hybrid structure with two sets of diffraction peaks at 2*θ* = 40.9°, 43.2°, 45.4°, 72.7°, 77.5° and 44.0°, 51.3°, 75.9°, which are assigned to the (221), (310), (311), (510), (520) planes of orthorhombic Ni_3_Mo_3_N (JCPDS 49-1336) [40, 41] and the (111), (200), (220) planes of face-centered cubic (fcc) Ni phase (JCPDS 04-0850) [36, 39], respectively (Fig. 1b). The diffraction peaks of Ni_3_Mo_3_N phase in nanoporous (NiFeCo)_3_(MoCr)_3_N/HEA shift to higher angles relative to the standard line patterns (Fig. 1c), indicating the incorporation of Fe, Co, and Cr in the Ni_3_Mo_3_N lattice by partially substituting the sublattice Ni sites and Mo sites to form complex (NiFeCo)_3_(MoCr)_3_N [42–44], respectively, in view of their similar atomic radius and bonding energies. While for the Ni phase in nanoporous (NiFeCo)_3_(MoCr)_3_N/HEA, the diffraction peaks shift to lower angles relative to the line patterns of fcc Ni as a consequence of incorporation of Cr and Mo with large atomic radius in addition to Fe and Co with similar atomic radius [45, 46]. Figure 1d shows a representative SEM image of nanoporous (NiFeCo)_3_(MoCr)_3_N/HEA electrode, where the island-like (NiFeCo)_3_(MoCr)_3_N precipitates with a width of ~ 200 nm are anchored on nanoporous HEA skeleton. This microstructural feature is also demonstrated by SEM-EDS elemental mappings of nanoporous (NiFeCo)_3_(MoCr)_3_N/HEA electrode (Fig. 1e), where the precipitated nanoislands are enriched in N element while Ni, Fe, Co, Mo, Cr, and Al atoms are uniformly distributed in both (NiFeCo)_3_(MoCr)_3_N nanoislands and HEA skeleton. Figure 1f shows a typical high-resolution TEM (HRTEM) image of (NiFeCo)_3_(MoCr)_3_N/HEA heterostructure interface, where both phases are identified by their corresponding fast Fourier transform (FFT) patterns (Fig. 1g, h). The interplanar spacings of ~ 0.218 and ~ 0.208 nm correspond to the lattice planes of orthorhombic (NiFeCo)_3_(MoCr)_3_N (221) and fcc HEA (111), deviating from the standard values of Ni_3_Mo_3_N (221) (0.222 nm) [40, 41] and Ni (111) (0.203 nm) [36, 39], respectively. Nevertheless, there does not observe evident interface viewed along their < 110 > and < 011 > zone axis, demonstrating the seamless integration of (NiFeCo)_3_(MoCr)_3_N on HEA skeleton. For comparison, nanoporous Ni_3_Mo_3_N/Ni, N-NiFeCo/NiFeCo and N-Ni/Ni with similar structure morphology are also prepared by the same alloying/dealloying and annealing procedures from their corresponding precursor alloys (Figs. S1, S2 and S6, S7).

**Fig. 2:**
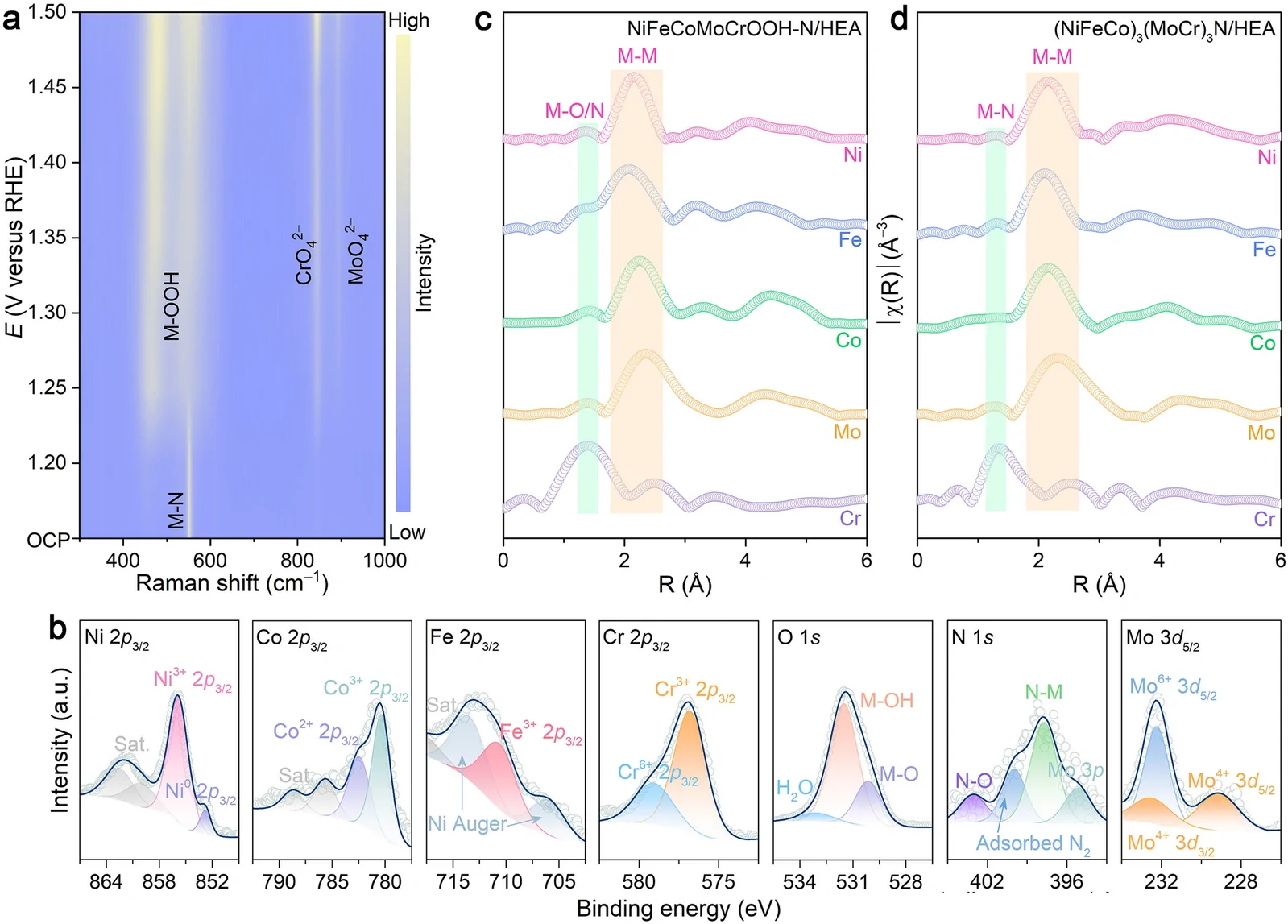
Chemical characterization of nanoporous multicomponent electrode. **a** Contour plots of in-situ Raman spectra of nanoporous (NiFeCo)_3_(MoCr)_3_N/HEA electrode at various applied potentials in 1 M KOH.** b** High-resolution Ni 2*p*_3/2_, Co 2*p*_3/2_, Fe 2*p*_3/2_, Cr 2*p*_3/2_, O 1*s*, N 1*s* and Mo 3*d*_5/2_ XPS spectra of nanoporous (NiFeCo)_3_(MoCr)_3_N/HEA after phase reconstruction. **c**, **d** FT-EXAFS spectra for Ni, Fe, Co, Mo and Cr elements of nanoporous (NiFeCo)_3_(MoCr)_3_N/HEA after (**c**) and before (**d**) phase reconstruction

In view of thermodynamically less stability of (NiFeCo)_3_(MoCr)_3_N [47–50], it undergoes phase transformation into amorphous NiFeCoMoCrOOH-N grafted on HEA when the nanoporous (NiFeCo)_3_(MoCr)_3_N/HEA working as the alkaline OER electrocatalyst (Fig. S8). Meanwhile, there also involves partial dissolution of Mo and Cr to generate MoO_4_^2−^ and CrO_4_^2−^ Brønsted basic oxyanions during the surface reconstructure (Fig. S9), of which the concentration increases and then reaches the plateau value within initial 25 min (Fig. S10). These electrochemical reconstruction processes are revealed by in-situ Raman spectroscopy characterization at various applied potentials (Fig. S11). Different from the Raman spectrum at open circuit potential (OCP), there appear neoformative Raman bands at ~ 470 cm^−1^ and ~ 550 cm^−1^ attributed to the *E*_g_ bending and *A*_1g_ stretching vibration modes of M-OOH [17, 51], respectively, at the applied potentials of > 1.2 V (Fig. 2a), verifying the formation of amorphous multicomponent oxyhydroxide of NiFeCoMoCrOOH-N. Owing to the N doping, the characteristic Raman bands of M-OOH in NiFeCoMoCrOOH-N display evident blueshifts relative to the ones in NiFeCoMoCrOOH that form on HEA during the initial OER of nanoporous (NiFeCo)_2_(MoCr)_3_O_8_/HEA electrode (Fig. S12). In addition, there also successively emerge the characteristic Raman bands of CrO_4_^2−^ (~ 845 cm^−1^) [52] at > 1.2 V and additional MoO_4_^2−^ (~ 891 cm^−1^) [18, 53] at > 1.25 V (Fig. 2a), respectively, due to the partial dissolution of Mo and Cr atoms from nanoporous (NiFeCo)_3_(MoCr)_3_N/HEA. As the applied potential increases from 1.2 to 1.5 V, these characteristic Raman bands gradually intensify, indicating more and more MoO_4_^2−^ and CrO_4_^2−^ anions electrostatically aggregating around the electrode during the water oxidation.

Figure 2b shows high-resolution Ni 2*p*_3/2_, Co 2*p*_3/2_, Fe 2*p*_3/2_, Cr 2*p*_3/2_, Mo 3*d*_5/3_, O 1*s*, and N 1*s* XPS spectra of NiFeCoMoCrOOH-N/HEA. Owing to the formation of NiFeCoMoCrOOH-N, all metal elements in the surface layer are primarily in the oxidized states of Ni^3+^, Co^2+^/Co^3+^, Fe^3+^, Cr^3+^/Cr^6+^ and Mo^4+^/Mo^6+^ species, in contrast with those in pristine nanoporous (NiFeCo)_3_(MoCr)_3_N/HEA, where they are in mixed metal states and low-valence oxidized states (Fig. S13). As for the N 1*s* XPS spectrum of NiFeCoMoCrOOH-N/HEA, the peaks at 397.8 and 403.1 eV are assigned to N-M and N-O, respectively, confirming that the doped N atoms bond with either metal and oxygen atoms [22, 54], in addition to the one at 400.0 eV corresponding to the adsorbed N_2_. The O 1*s* XPS spectrum shows three oxygen species, including lattice oxygen (M-O), absorbed OH (M-OH) and adsorbed H_2_O [27, 54]. In particular, the peak of M-O in NiFeCoMoCrOOH-N/HEA displays a positive shift relative to that in NiMoOOH-N/Ni (Fig. S14), unveiling a higher lattice oxygen activity in N-doped NiFeCoMoCrOOH due to the incorporation of Fe, as well as Co and Cr [27, 51].

The local atomic and electronic structures of NiFeCoMoCrOOH-N/HEA are also investigated by X-ray absorption near-edge structure (XANES) and extended X-ray absorption fine structure (EXAFS). In the Ni, Fe, Co, Mo, and Cr K-edge XANES spectra (Fig. S15), the NiFeCoMoCrOOH-N/HEA has the near-edge energies to locate between (NiFeCo)_3_(MoCr)_3_N/HEA and their reference oxides, demonstrating their different accumulation of electrons on transition metal atoms as a result of the formation M-O/N coordination [21, 55, 56]. Figure 2c shows the Fourier-transformed EXAFS (FT-EXAFS) spectra of Ni, Fe, Co, Mo, and Cr K-edge in NiFeCoMoCrOOH-N/HEA. All transition metals in the first and second shells are of similar radial distances and amplitudes, suggesting that they are in consistent local atomic environments of M-O/N and M-M coordination due to full random mixing distribution of transition metal atoms in NiFeCoMoCrOOH-N and HEA [56–58]. These M-O/N bond lengths are slightly elongated or shortened relative to those in the (NiFeCo)_3_(MoCr)_3_N/HEA (Fig. 2d) or in the NiFeCoMoCrOOH without N doping (Fig. S16). Compared with the Ni-O/N and Mo-O/N in NiMoOOH-N, the incorporation of Fe, Co, and Cr in NiFeCoMoCrOOH-N elongates their bond lengths.

### Electrochemical Characterizations of Nanoporous Electrodes

**Fig. 3:**
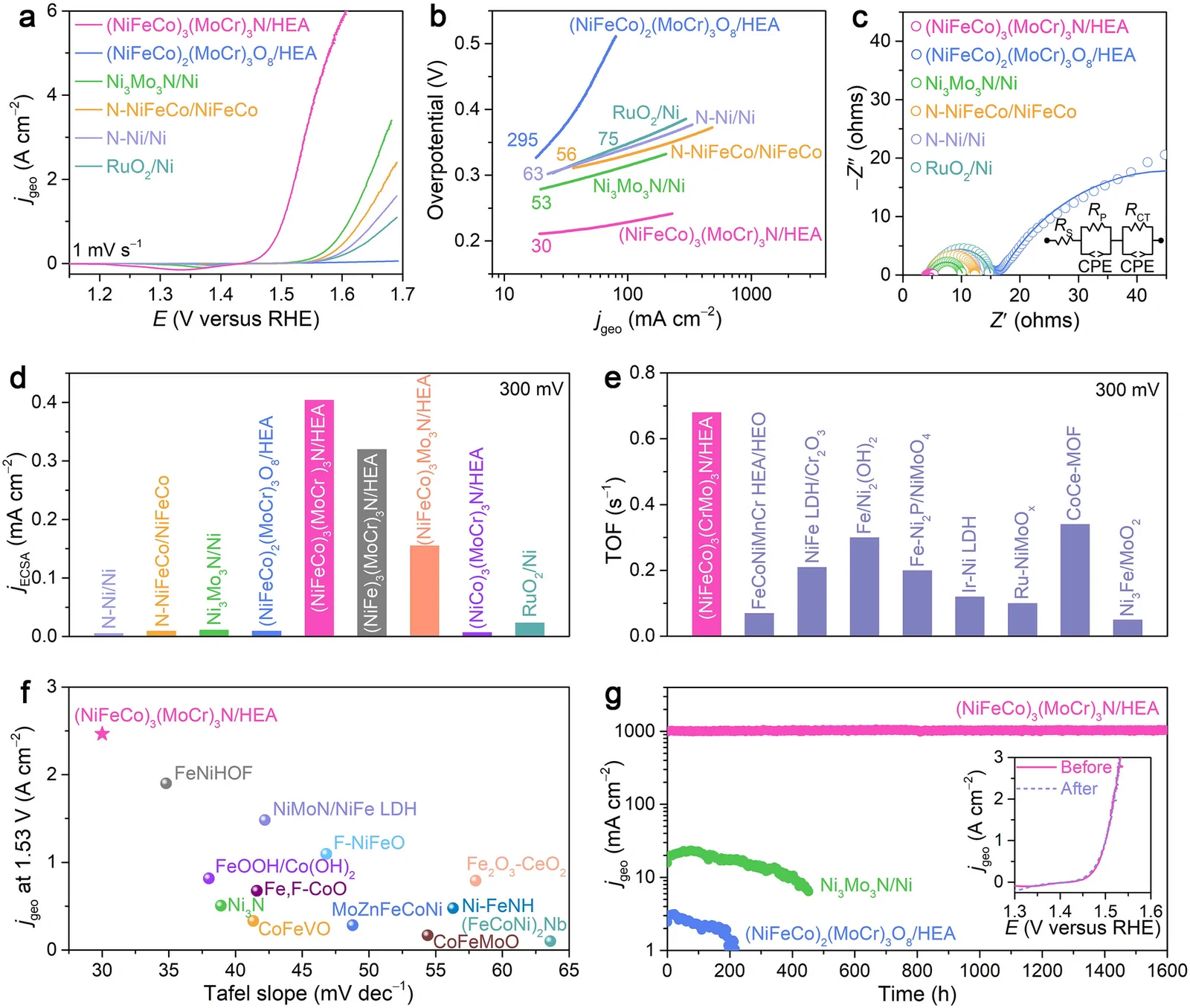
Alkaline OER electrocatalytic properties.** a** OER polarization curves for nanoporous (NiFeCo)_3_(MoCr)_3_N/HEA, Ni_3_Mo_3_N/Ni, (NiFeCo)_2_(MoCr)_3_O_8_/HEA, N-NiFeCo/NiFeCo and N-Ni/Ni electrodes, as well as commercially available RuO_2_ catalysts immobilized on nanoporous Ni (RuO_2_/Ni) in 1 M KOH electrolyte. Scan rate: 1 mV s^−1^. **b** Tafel slopes for different nanoporous electrodes according to the OER polarization curves in (**a**). **c** EIS spectra of nanoporous (NiFeCo)_3_(MoCr)_3_N/HEA, Ni_3_Mo_3_N/Ni, (NiFeCo)_2_(MoCr)_3_O_8_/HEA, N-NiFeCo/NiFeCo, N-Ni/Ni and RuO_2_/Ni electrodes. Inset: the electrical equivalent circuit.** d** Comparison of specific activities for nanoporous (NiFeCo)_3_(MoCr)_3_N/HEA, (NiFe)_3_(MoCr)_3_N/HEA, (NiCo)_3_(MoCr)_3_N/HEA, (NiFeCo)_3_Mo_3_N/HEA, Ni_3_Mo_3_N/Ni, (NiFeCo)_2_(MoCr)_3_O_8_/HEA, N-NiFeCo/NiFeCo, N-Ni/Ni and RuO_2_/Ni at the overpotential of 300 mV. **e** TOF value of nanoporous (NiFeCo)_3_(MoCr)_3_N/HEA at the overpotential of 300 mV, comparing with those of representative OER electrocatalysts reported previously. **f** Tafel slope and current density of nanoporous (NiFeCo)_3_(MoCr)_3_N/HEA at the potential of 1.53 V versus RHE, comparing with the values of nonprecious metal-based OER electrocatalysts operating under high-current density reported recently. **g** Long-term stability of nanoporous (NiFeCo)_3_(MoCr)_3_N/HEA electrode at the overpotential of 270 mV for 1600 h, comparing with those of nanoporous Ni_3_Mo_3_N/Ni and (NiFeCo)_2_(MoCr)_3_O_8_/HEA electrodes at the same overpotentials for 450 h and 200 h, respectively. Inset: polarization curves before and after the durability measurement of nanoporous (NiFeCo)_3_(MoCr)_3_N/HEA electrode

Figure 3a shows the LSV curve of nanoporous (NiFeCo)_3_(MoCr)_3_N/HEA in 1 M KOH electrolyte, comparing with those of nanoporous (NiFeCo)_2_(MoCr)_3_O_8_/HEA, Ni_3_Mo_3_N/Ni, N-NiFeCo/NiFeCo and N-Ni/Ni, as well as commercially available RuO_2_ catalysts immobilized on nanoporous Ni current collector (RuO_2_/Ni). Here the potentials are calibrated with respect to the reversible hydrogen electrode (RHE) according to the equation: *E*_RHE_ = *E*_Ag/AgCl_ + 1.016 V in 1 M KOH (Fig. S17). Evidently, the nanoporous (NiFeCo)_3_(MoCr)_3_N/HEA electrode exhibits superior OER electrocatalysis to deliver current density of as high as ~ 2.5 A cm^−2^ at low overpotential of 300 mV, much higher than the value required for industrial H_2_ production via water electrolysis (> 0.5 A cm^−2^) [9, 10, 31]. As the overpotential increases to 370 mV, the OER current density dramatically increases to ~ 6 A cm^−2^, in sharp contrast to those of nanoporous (NiFeCo)_2_(MoCr)_3_O_8_/HEA (~ 0.03 A cm^−2^), Ni_3_Mo_3_N/Ni (~ 0.74 A cm^−2^), N-NiFeCo/NiFeCo (~ 0.45 A cm^−2^) and N-Ni/Ni (~ 0.27 A cm^−2^) electrodes. This value is ~ 30-fold higher than that of RuO_2_/Ni (~ 0.2 A cm^−2^) although RuO_2_ is generally considered as one of the most efficient OER electrocatalysts [9, 12, 14]. The enhanced reaction kinetics of nanoporous (NiFeCo)_3_(MoCr)_3_N/HEA electrode is also reflected by its ultralow Tafel slope of ~ 30 mV dec^−1^, the smallest value among the nanoporous electrodes (Fig. 3b). Electrochemical impedance spectroscopy analysis further demonstrates the superior kinetics of nanoporous (NiFeCo)_3_(MoCr)_3_N/HEA for OER electrocatalysis. In the Nyquist plot (Fig. 3c), its EIS spectrum exhibits two characteristic semicircles in the middle- to low-frequency range, which correspond to the charge transfer resistance (*R*_CT_) and the pore resistance (*R*_P_) in parallel with the constant phase elements (CPEs) [36, 39]. The intercept at high frequencies on the real axis reflects the series resistance (*R*_S_) of both electrolyte and electrode [32, 54]. Based on the equivalent circuit (inset of Fig. 3c), the *R*_CT_ value of nanoporous (NiFeCo)_3_(MoCr)_3_N/HEA electrode is determined to be as low as ~ 1.1 Ω, much lower than those of nanoporous Ni_3_Mo_3_N/Ni (~ 5.6 Ω), (NiFeCo)_2_(MoCr)_3_O_8_/HEA (~ 58.2 Ω), N-NiFeCo/NiFeCo (~ 7.3 Ω) and N-Ni/Ni (~ 8.3 Ω) as well as RuO_2_/Ni electrodes (~ 10.4 Ω) (Fig. S18). Figure S19 presents the OER polarization curves of nanoporous (NiFeCo)_3_(MoCr)_3_N/HEA with different levels of iR compensation based on the *R*_S_ value, which is repeatably measured on eight samples (Fig. S20). Even without iR compensation, it achieves a current density of ~ 2.5 A cm^–2^ at overpotential of ~ 707 mV, demonstrating its outstanding ampere-level OER catalytic activity.

In view that these investigated nanoporous electrodes are of similar pore architecture to facilitate electron transfer and mass transport (Figs. 1d and S7), their relative intrinsic activities (*j*_ECSA_) at the overpotential of 300 mV are evaluated according to the ECSAs, which are determined by the double-layer capacitances derived from CV curves in the non-Faradaic potential window of − 0.1 to 0 V (versus Ag/AgCl) (Fig. S21 and Note S1). Owing to the presence of NiFeCoMoCrOOH-N active phase, the nanoporous (NiFeCo)_3_(MoCr)_3_N/HEA exhibits the highest intrinsic activity with *j*_ECSA_ =  ~ 0.404 mA cm_ECSA_^−2^ (Fig. 3d). This value is ~ 40-fold higher than that of nanoporous (NiFeCo)_2_(MoCr)_3_O_8_/HEA (~ 0.009 mA cm_ECSA_^−2^), implying the significant role of N in modulating adsorption energies of active sites in multicomponent oxyhydroxide to accelerate OER kinetics. In contrast, the *j*_ECSA_ value of nanoporous Ni_3_Mo_3_N/Ni is as low as ~ 0.011 mA cm_ECSA_^−2^, ~ 35-fold lower than that of nanoporous (NiFeCo)_3_(MoCr)_3_N/HEA. Such dramatic difference reflects the synergic effect of Fe, Co and Cr on the intrinsic activity of N-doped multicomponent oxyhydroxide. In order to reveal the individual role of Fe, Co or Cr, nanoporous (NiFe)_3_(MoCr)_3_N/HEA, (NiCo)_3_(MoCr)_3_N/HEA and (NiFeCo)_3_Mo_3_N/HEA electrodes are prepared by the same alloying/dealloying and annealing procedures based on their precursor alloys without the incorporation of Co, Fe, or Cr (Figs. S22–S26). Owing to the absence of Fe, Co, or Cr component, they encounter more or less reduction in OER electrocatalysis compared with nanoporous (NiFeCo)_3_(MoCr)_3_N/HEA (Fig. S27). Intriguingly, the *j*_ECSA_ of nanoporous (NiCo)_3_(MoCr)_3_N/HEA is as low as ~ 0.007 mA cm_ECSA_^−2^, reflecting that the Fe in multicomponent oxyhydroxide acts as a critical active site to mediate the alkaline OER. While for the nanoporous N-NiFeCo/NiFeCo electrode, it exhibits a *j*_ECSA_ of ~ 0.009 mA cm_ECSA_^−2^ probably due to the absence of Brønsted basic oxyanions of MoO_4_^2−^ and CrO_4_^2−^ around electrode surface. In contrast, the concentrations of MoO_4_^2−^ and CrO_4_^2−^ around nanoporous (NiFeCo)_3_(MoCr)_3_N/HEA electrode reach as high as 13.7 and 35.3 µg cm^−2^, respectively (Fig. S28 and Note S2), which enable the formation of cooperative solid-molecular interface to configure multiple active sites to further boost the OER kinetics. The MoO_4_^2−^ and CrO_4_^2−^ anions serving as the molecular active sites is also demonstrated by dramatic electrocatalysis degradation of nanoporous (NiFeCo)_3_(MoCr)_3_N/HEA in fresh KOH electrolyte without MoO_4_^2−^ and CrO_4_^2−^ anions compared with in original one with MoO_4_^2−^ and CrO_4_^2−^ (Fig. S29). Similar phenomenon is also observed for Mo/Cr-free nanoporous N-NiFeCo/NiFeCo electrode, which exhibits much higher OER activity in KOH containing both MoO_4_^2−^ and CrO_4_^2−^ than individual MoO_4_^2−^ or CrO_4_^2−^, as well as in pure KOH (Fig. S30), indicating the MoO_4_^2−^ and CrO_4_^2−^ as the molecular co-catalysts to accelerate OER kinetics. These observations indicate the synergic effect of multicomponent oxyhydroxide solid active sites and Brønsted basic molecular active sites in boosting the OER kinetics on nanoporous (NiFeCo)_3_(MoCr)_3_N/HEA, which is also reflected by the turnover frequency (TOF) of as high as ~ 0.68 s^−1^ (Fig. 3e and Note S3). These impressive electroactive properties enlist nanoporous (NiFeCo)_3_(MoCr)_3_N/HEA as an attractive OER electrocatalyst to outperform some of the best nonprecious metal-based OER electrocatalysts reported recently (Fig. 3f and Table S1) [9, 10, 14–16, 24, 26, 27, 31]. To demonstrate the reproducibility, more eight nanoporous (NiFeCo)_3_(MoCr)_3_N/HEA electrodes are fabricated by the same alloying/dealloying and annealing procedures. They display the almost overlapping OER polarization curves (Fig. S31), indicating the excellent reproducibility.

To investigate the OER stability of nanoporous (NiFeCo)_3_(MoCr)_3_N/HEA electrode, the continuous electrolysis measurement is performed at the overpotential of 270 mV for > 1600 h in 1 M KOH electrolyte at room temperature. As shown in Fig. 3g, there does not observe evident fluctuation at the ampere-level current density, attesting the exceptional durability of nanoporous (NiFeCo)_3_(MoCr)_3_N/HEA electrode. After the long-term stability measurement, it still displays an alkaline OER polarization curve almost overlapping with the original one (inset of Fig. 3g) and maintains the initial microstructure composed of uniform N-doped NiFeCoMoCrOOH nanosheets on HEA skeleton (Fig. S32) with similar chemical states (Fig. S33). The exceptional stability of nanoporous (NiFeCo)_3_(MoCr)_3_N/HEA is also demonstrated by ICP-OES analysis on the tested electrolyte, where only trace Ni, Fe and Co are detected in addition to Mo and Cr ions forming a locally microenvironment to enhance the OER kinetics (Table S2). In stark contrast, nanoporous Ni_3_Mo_3_N/Ni and (NiFeCo)_2_(MoCr)_3_O_8_/HEA electrodes suffer from dramatic current density reduction only in 450 and 200 h, respectively.

### Mechanism Investigation

**Fig. 4:**
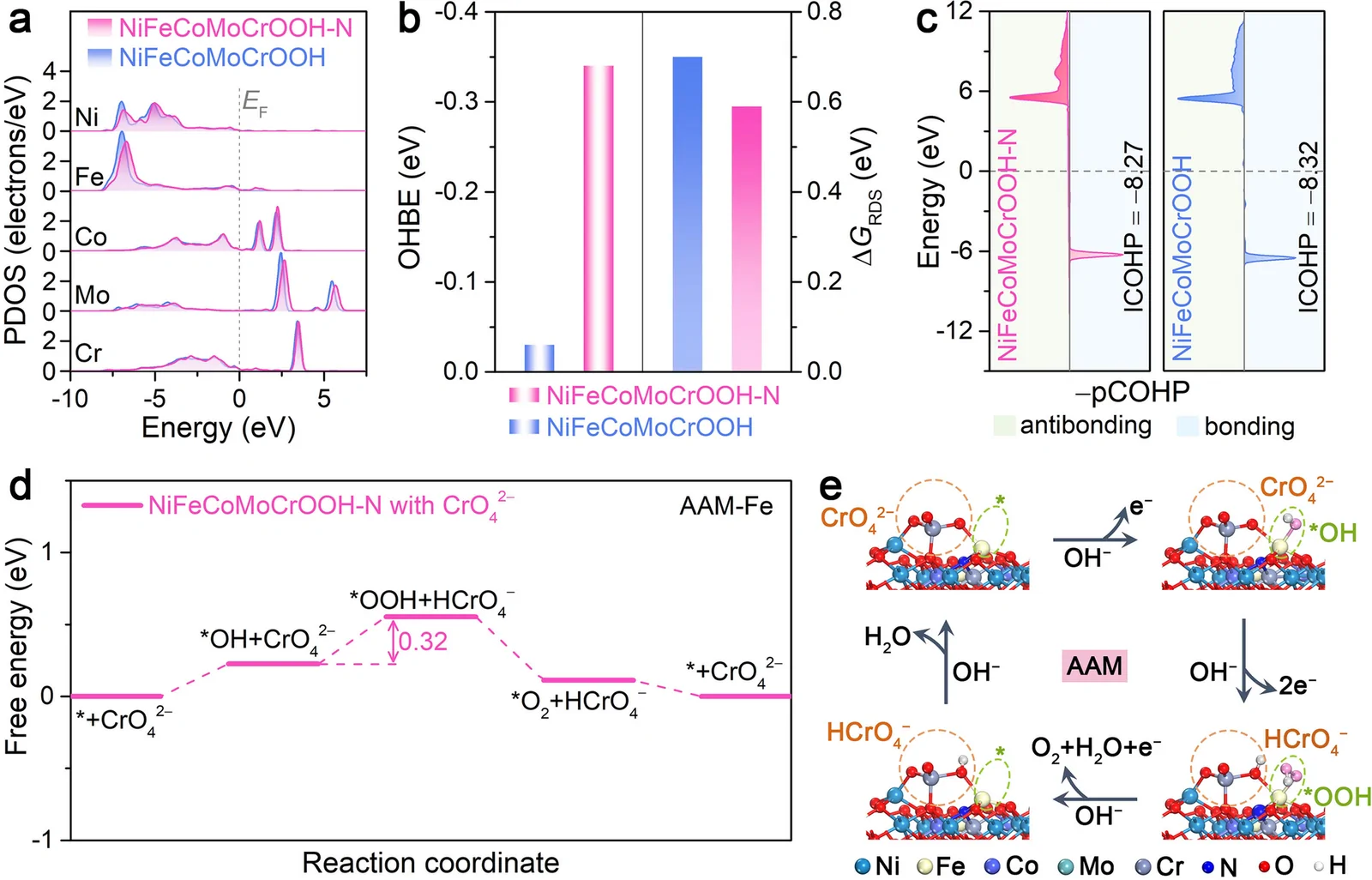
Mechanism investigations. **a** Projected density of states (PDOS) of Ni, Fe, Co, Mo and Cr in NiFeCoMoCrOOH-N and NiFeCoMoCrOOH. **b** OHBE and Δ*G*_RDS_ values for the alkaline OER mediated by NiFeCoMoCrOOH, NiFeCoMoCrOOH-N. **c** ICOHP values of O-H bond for the adsorbed *OH on surface Fe site of NiFeCoMoCrOOH-N and NiFeCoMoCrOOH. **d** Gibbs free energy diagram of alkaline OER on NiFeCoMoCrOOH-N with CrO_4_^2−^ following AAM path. **e** Interface configurations of NiFeCoMoCrOOH-N with CrO_4_^2−^ at four different stages following AAM path

To theoretically illustrate the OER electrocatalytic mechanism, DFT calculations are conducted on NiFeCoMoCrOOH and NiFeCoMoCrOOH-N without/with CrO_4_^2−^ (Fig. S34). For the NiFeCoMoCrOOH, it thermodynamically prefers to mediate the alkaline OER via the AEM pathway by making use of surface Fe atoms as the optimal active sites (Fig. S35). Consequently, the NiFeCoMoCrOOH-N is constructed by N atom substituting Fe-coordinated O atom for further DFT simulations, where the surface Fe atoms are investigated as the active sites. Figure 4a shows the projected density of states (PDOS) of Ni, Fe, Co, Mo and Cr atoms of NiFeCoMoCrOOH-N, displaying evident upshifts of *d* bands relative to those of NiFeCoMoCrOOH. This is because the N doping causes electronic redistributions to enhance adsorption energies of metal atoms in NiFeCoMoCrOOH-N. As shown in Fig. S36 for the PDOS of *OH adsorbed on Fe atom, their strong *p*-*d* orbital hybridization results in a *OH binding energy (OHBE) of − 0.34 eV, much stronger than that of NiFeCoMoCrOOH (− 0.03 eV) (Fig. 4b). In the differential charge density mapping of *OH adsorbed on Fe site of NiFeCoMoCrOOH-N (inset of Fig. S36), there appears evident electron accumulation on O of *OH and depletion around Fe atom, which activates the O-H bond for deprotonation. The crystal orbital Hamilton population (COHP) analysis of O-H bond in the *OH reveals a more positive integral COHP (ICOHP) value on Fe site of NiFeCoMoCrOOH-N compared with that of NiFeCoMoCrOOH (Fig. 4c), suggesting the enhanced deprotonation reaction kinetics. Figure S37 shows the reaction Gibbs free energy (Δ*G*) profiles for the alkaline OER in AEM pathway on NiFeCoMoCrOOH-N and NiFeCoMoCrOOH, where both solid catalysts suffer from the same rate-determining step (RDS), i.e., the deprotonation of *OH to form *O. The Δ*G*_RDS_ value decreases from 0.70 eV on the NiFeCoMoCrOOH to 0.59 eV on the NiFeCoMoCrOOH-N (Fig. 4b), confirming the significant role of N in modulating electrocatalytic activity of multicomponent oxyhydroxide.

The electrostatic accumulation of MoO_4_^2−^ and CrO_4_^2−^ Brønsted basic oxyanions on NiFeCoMoCrOOH-N solid surface gives rise to the formation of solid-molecular interfaces to configure unconventional multiple active sites, which mediate the OER in an anion-assisted mechanism (AAM) pathway by making use of MoO_4_^2−^ and CrO_4_^2−^ anions act as molecular co-catalysts. Considering that the CrO_4_^2−^ has a higher concentration near solid NiFeCoMoCrOOH-N surface than the MoO_4_^2−^ (Fig. S28), the CrO_4_^2−^ is selected as the representative species during the DFT simulation for simplicity. Figure 4d shows the reaction Gibbs free energy profile for the OER on NiFeCoMoCrOOH-N with CrO_4_^2−^ anions (Note S4). Different from the AEM pathway on NiFeCoMoCrOOH-N (Fig. S34), the CrO_4_^2−^ anions participate the PCET processes in AAM route, breaking the LSRs of multiple oxygen intermediates to accelerate reaction kinetics. By virtue of the chemical feature of Brønsted basic oxyanions, there takes place *H intermediate spillover from the contiguous *OH on Fe site to CrO_4_^2−^ to form HCrO_4_^−^ specie. Meanwhile, the *O intermediate is attacked by OH^−^ to generate *OOH intermediate on Fe site for subsequent reaction with OH^−^ to release O_2_, followed by the final deprotonation of HCrO_4_^−^ to regenerate the CrO_4_^−^ (Fig. 4e). Therein, the step of *OH deprotonation and CrO_4_^2−^ protonation to form *O and HCrO_4_^−^ via *H spillover acts as the RDS in the AAM pathway, with the Δ*G*_RDS_ of as low as 0.32 eV (Fig. 4d), which aligns with the Tafel slope during the OER. This AAM pathway is experimentally demonstrated by pH dependence measurements. Owing to the NiFeCoMoCrOOH-N/CrO_4_^2−^ solid-molecular interfaces, which accelerates deprotonation of OH^−^ and protonation of CrO_4_^2−^ via the *H spillover, the nanoporous (NiFeCo)_3_(MoCr)_3_N/HEA displays a stronger pH dependence of OER current density relative to the nanoporous N-NiFeCo/NiFeCo (Fig. S38) [17, 24, 59].

### Electrolyzer Performance

**Fig. 5:**
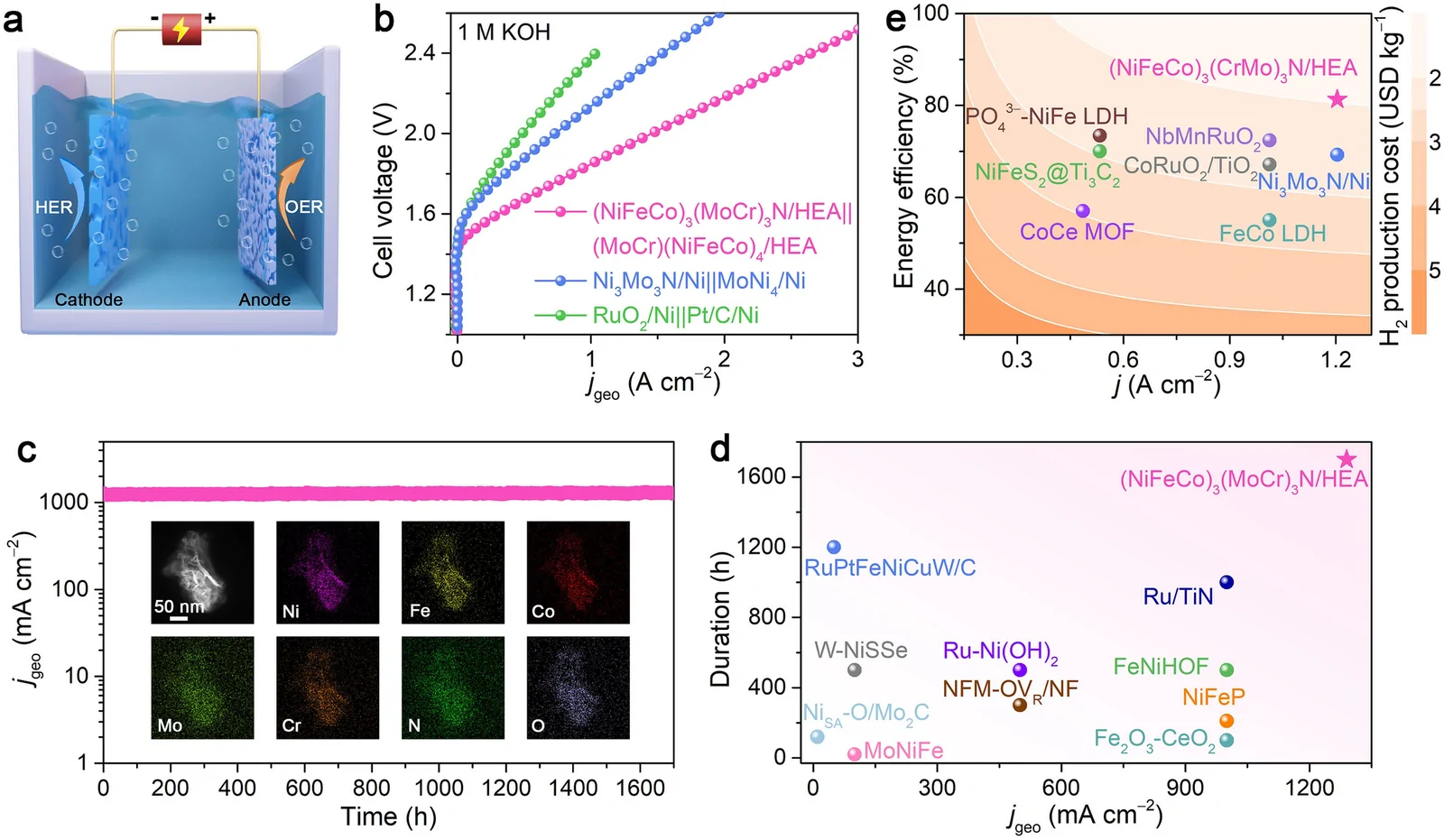
Alkaline water electrolyzer performance. **a** Schematic illustration of alkaline water electrolyzer that is constructed with nanoporous (NiFeCo)_3_(MoCr)_3_N/HEA anode and nanoporous (MoCr)(NiFeCo)_4_/HEA cathode in 1 M KOH electrolyte. **b** Typical polarization curves of (NiFeCo)_3_(MoCr)_3_N/HEA||(MoCr)(NiFeCo)_4_/HEA, Ni_3_Mo_3_N/Ni||MoNi_4_/Ni and RuO_2_/Ni||Pt/C/Ni electrolyzers for alkaline water electrolysis. Electrolyte: 1 M KOH. **c** Durability of (NiFeCo)_3_(MoCr)_3_N/HEA||(MoCr)(NiFeCo)_4_/HEA electrolyzer at 1.92 V for 1700 h. Inset: STEM image of (NiFeCo)_3_(MoCr)_3_N/HEA electrode and its corresponding EDS elemental mappings of Ni, Fe, Co, Mo, Cr, N and O after the durability measurement for 1700 h. **d** Current density and durability test time of nanoporous (NiFeCo)_3_(MoCr)_3_N/HEA-based electrolyzer, comparing with the values of representative alkaline water electrolyzers reported previously. **e** Comparison of energy efficiency and H_2_ production cost for (NiFeCo)_3_(MoCr)_3_N/HEA-based electrolyzer with the values of other advanced electrolyzers reported recently

To demonstrate the practical use in water electrolysis for hydrogen production, (NiFeCo)_3_(MoCr)_3_N/HEA||(MoCr)(NiFeCo)_4_/HEA AWE cell is constructed with nanoporous (NiFeCo)_3_(MoCr)_3_N/HEA as the anode and nanoporous (MoCr)(NiFeCo)_4_/HEA as the cathode (Fig. S39) in 1 M KOH (Fig. 5a). As shown in Fig. 5b, the (NiFeCo)_3_(MoCr)_3_N/HEA||(MoCr)(NiFeCo)_4_/HEA AWE can achieve ampere-level current densities at the cell voltage of ~ 1.85 V, much lower than the one that is assembled with nanoporous Ni_3_Mo_3_N/Ni and MoNi_4_/Ni (Ni_3_Mo_3_N/Ni||MoNi_4_/Ni) (~ 2.13 V), or commercially available RuO_2_ and Pt/C immobilized on nanoporous Ni (RuO_2_/Ni||Pt/C/Ni) (~ 2.38 V) (Fig. 5b). When operating at the cell voltage of 1.92 V without iR compensation, the (NiFeCo)_3_(MoCr)_3_N/HEA||(MoCr)(NiFeCo)_4_/HEA AWE always maintains stable current density at ~ 1.25 A cm^−2^ with ~ 1.4% fluctuation for over 1700 h to continuously generate hydrogen gas (Fig. 5c). During the long-term operation of AWE for 1700 h, the nanoporous (NiFeCo)_3_(MoCr)_3_N/HEA electrode still exhibits impressive stability, as illustrated by uniform distribution of Ni, Fe, Co, Mo, Cr, N, and O atoms in STEM-EDS element mapping images of NiFeCoMoCrOOH-N nanosheets after water electrolysis. Two more (NiFeCo)_3_(MoCr)_3_N/HEA||(MoCr)(NiFeCo)_4_/HEA AWEs also display stable current density at 1.92 V (Fig. S40), indicating the outstanding reproducibility. These excellent electrochemical performances enlist the AWE based on (NiFeCo)_3_(MoCr)_3_N/HEA electrode to outperform some of the best ones reported previously (Fig. 5d and Table S3) [9, 10, 14, 16, 20, 26, 31]. The H_2_ production cost of the (NiFeCo)_3_(MoCr)_3_N/HEA-based AWE is estimated to be ~ 1.98 USD kg_H2_^−1^ (Note S5) with a high energy efficiency of 81.3% (Note S6), satisfying the 2030 target (2.0–2.5 USD kg_H2_^−1^) proposed by the U.S. Department of Energy (DOE), which demonstrates the AWE is more economically competitive than other advanced electrolyzers (Fig. 5e) [16, 17, 20, 26, 30, 60].

## Conclusions

In summary, we have developed nonprecious metal-based multicomponent nitride/alloy heterostructure electrode, which is composed of (NiFeCo)_3_(MoCr)_3_N complex nitride integrated on HEA skeleton, as a versatile OER electrocatalyst for ampere-level alkaline water oxidation. Under strongly oxidation environment, the constituent (NiFeCo)_3_(MoCr)_3_N undergoes in-situ electrochemical phase reconstruction into amorphous NiFeCoMoCrOOH-N nanosheets along with the partial dissolution of Mo and Cr atoms to generate MoO_4_^2−^ and CrO_4_^2−^ Brønsted basic anions, which electrostatically aggregate on solid NiFeCoMoCrOOH-N surface to configure cooperative solid-molecular interfaces with multiple active sites. During the alkaline OER, the doped N atom modulates the surface Fe-O coordination in the solid multicomponent oxyhydroxide to enable *OH adsorption and deprotonation on Fe sites, followed by *H spillover to the contiguous MoO_4_^2−^ and CrO_4_^2−^ for protonation to facilitate the formation of *OOH with low energy barrier. This unconventional molecule-assisted multi-site mechanism accelerates the alkaline OER kinetics. The intrinsic activity reaches as high as ~ 0.404 mA cm_ECSA_^−2^ at the overpotential of 300 mV. By virtue of unique architecture that endows abundant active sites and facilitates electron transfer and reactant species transportation, the nanoporous (NiFeCo)_3_(MoCr)_3_N/HEA electrode exhibits exceptional OER electrocatalysis to deliver ultrahigh current density of ~ 2.5 A cm^−2^ at low overpotential of 300 mV. In a long-term durability test at the overpotential of 270 mV with ampere-level current density, it maintains exceptional stability. These electrocatalytic properties enlist (NiFeCo)_3_(MoCr)_3_N/HEA||(MoCr)(NiFeCo)_4_/HEA AWE to reach 1.25 A cm^−2^ at a low cell voltage of 1.92 V and stably operate over 1700 h for continuous hydrogen production. This work demonstrates developing multicomponent catalysts as an effective strategy to mediate multiple-intermediate reaction via an unconventional molecule-assisted multi-site mechanism.

## Supplementary Information

Below is the link to the electronic supplementary material.

Supplementary file1 (DOCX 19373 kb)

## References

[1] T. Terlouw, C. Moretti, C. Harpprecht, R. Sacchi, R. McKenna et al., Global greenhouse gas emissions mitigation potential of existing and planned hydrogen projects. Nat. Energy **10**, 1503–1515 (2025). 10.1038/s41560-025-01892-9

[2] C. Gunathilake, I. Soliman, D. Panthi, P. Tandler, O. Fatani et al., A comprehensive review on hydrogen production, storage, and applications. Chem. Soc. Rev. **53**(22), 10900–10969 (2024). 10.1039/d3cs00731f 39421882

[3] K.D. Kleijne, M.A.J. Huijbregts, F. Knobloch, R.V. Zelm, J.P. Hilbers et al., Worldwide greenhouse gas emissions of green hydrogen production and transport. Nat. Energy **9**, 1139–1152 (2024). 10.1038/s41560-024-01563-1

[4] X.Q. Gao, Y.T. Chen, Y.J. Wang, L.Y. Zhao, X.Y. Zhao et al., Next-generation green hydrogen: progress and perspective from electricity, catalyst to electrolyte in electrocatalytic water splitting. Nano-Micro Lett. **16**, 237 (2024). 10.1007/s40820-024-01424-2 PMC11226619 38967856

[5] M. Muhyuddin, C. Santoro, L. Osmieri, V.C.A. Ficca, A. Friedman et al., Anion-exchange-membrane electrolysis with alkali-free water feed. Chem. Rev. **125**(15), 6906–6976 (2025). 10.1021/acs.chemrev.4c00466 40748968 PMC12355722

[6] D. Henkensmeier, W.C. Cho, P. Jannasch, J. Stojadinovic, Q. Li et al., Separators and membranes for advanced alkaline water electrolysis. Chem. Rev. **124**(10), 393–6443 (2024). 10.1021/acs.chemrev.3c00694 PMC11117188 38669641

[7] L. Wan, Z.A. Xu, Q. Xu, M.B. Pang, D.C. Lin et al., Key components and design strategy of the membrane electrode assembly for alkaline water electrolysis. Energy Environ. Sci. **16**(4), 1384–1480 (2023). 10.1039/d3ee00142c

[8] A. Odenweller, F. Ueckerdt, The green hydrogen ambition and implementation gap. Nat. Energy **10**, 110–123 (2025). 10.1038/s41560-024-01684-7

[9] L. Zhang, Q.L. Xu, L. Xia, W.Y. Jiang, K.W. Wang et al., Asymmetrically tailored catalysts towards electrochemical energy conversion with non-precious materials. Chem. Soc. Rev. **54**(10), 5108–5145 (2025). 10.1039/d4cs00710g 40277188

[10] S.J. Yang, X.H. Liu, S.S. Li, W.J. Yuan, L.N. Yang et al., The mechanism of water oxidation using transition metal-based heterogeneous electrocatalysts. Chem. Soc. Rev. **53**(11), 5593–5625 (2024). 10.1039/d3cs01031g 38646825

[11] W.J. Shi, T.H. Shen, C.K. Xing, K. Sun, Q.S. Yan et al., Ultrastable supported oxygen evolution electrocatalyst formed by ripening-induced embedding. Science **387**(6735), 791–796 (2025). 10.1126/science.adr3149 39946454

[12] Y. Park, H.Y. Jang, T.K. Lee, T. Kim, D. Kim et al., Atomic-level Ru-Ir mixing in rutile-type (RuIr)O_2_ for efficient and durable oxygen evolution catalysis. Nat. Commun. **16**, 579 (2025). 10.1038/s41467-025-55910-1 39794326 PMC11723980

[13] Z.Z. Wang, J.Q. Zhou, Y.R. Shi, L. Luo, H.R. Li et al., Multi-site electrocatalysts for hydrogen production under neutral conditions. Chem. Soc. Rev. **55**(1), 254–298 (2026). 10.1039/d5cs00881f 41221893

[14] J.T. Ren, L. Chen, H.Y. Wang, Z.Y. Yuan, High-entropy alloys in electrocatalysis: from fundamentals to applications. Chem. Soc. Rev. **52**(23), 8319–8373 (2023). 10.1039/d3cs00557g 37920962

[15] D.Y. Li, R. Xiang, F. Yu, J.S. Zeng, Y. Zhang et al., In situ regulating cobalt/iron oxide-oxyhydroxide exchange by dynamic iron incorporation for robust oxygen evolution at large current density. Adv. Mater. **36**(5), 2305685 (2024). 10.1002/adma.202305685 37747155

[16] Y.Z. Chen, Q.B. Li, Y.X. Lin, J. Liu, J. Pan et al., Boosting oxygen evolution reaction by FeNi hydroxide-organic framework electrocatalyst toward alkaline water electrolyzer. Nat. Commun. **15**, 7278 (2024). 10.1038/s41467-024-51521-4 39179616 PMC11344037

[17] J.Q. Wang, Y. Liu, G.C. Yang, Y.Q. Jiao, Y.M. Dong et al., MXene-assisted NiFe sulfides for high-performance anion exchange membrane seawater electrolysis. Nat. Commun. **16**, 1319 (2025). 10.1038/s41467-025-56639-7 39900925 PMC11790850

[18] Q. Zhang, W. Zhang, J.W. Zhu, X.Y. Zhou, G.R. Xu et al., Tuning d–p orbital hybridization of NiMoO_4_@Mo_15_Se_19_/NiSe_2_ core-shell nanomaterials via asymmetric coordination interaction enables the water oxidation process. Adv. Energy Mater. **14**(20), 2304546 (2024). 10.1002/aenm.202304546

[19] Z.P. Wu, S.W. Zuo, Z.H. Pei, J. Zhang, L.R. Zheng et al., Operando unveiling the activity origin via preferential structural evolution in Ni-Fe (oxy)phosphides for efficient oxygen evolution. Sci. Adv. **11**(10), eadu5370 (2025). 10.1126/sciadv.adu5370 40053602 PMC11887844

[20] A. Nairan, Z. Feng, R.M. Zheng, U. Khan, J.K. Gao, Engineering metallic alloy electrode for robust and active water electrocatalysis with large current density exceeding 2000 mA cm^−2^. Adv. Mater. **36**(29), 2401448 (2024). 10.1002/adma.202401448 38518760

[21] H.H. Hu, X.L. Wang, Z.R. Zhang, J.H. Liu, X.H. Yan et al., Engineered nickel-iron nitride electrocatalyst for industrial-scale seawater hydrogen production. Adv. Mater. **37**(4), 2415421 (2025). 10.1002/adma.202415421 39584400

[22] Y.W. Liu, L.R. Wang, R. Hübner, J. Kresse, X.M. Zhang et al., Cobalt-based Co_3_Mo_3_N/Co_4_N/Co metallic heterostructure as a highly active electrocatalyst for alkaline overall water splitting. Angew. Chem. Int. Ed. **63**(14), e202319239 (2024). 10.1002/anie.202319239 38314947

[23] X. Luo, H.Y. Zhao, X. Tan, S. Lin, Y.S. Yu et al., Fe-S dually modulated adsorbate evolution and lattice oxygen compatible mechanism for water oxidation. Nat. Commun. **15**, 8293 (2024). 10.1038/s41467-024-52682-y 39333518 PMC11436974

[24] Y.J. Mei, J.L. Chen, Q. Wang, Y.Q. Guo, H.W. Liu et al., MoZn-based high entropy alloy catalysts enabled dual activation and stabilization in alkaline oxygen evolution. Sci. Adv. **10**(47), eadq6758 (2024). 10.1126/sciadv.adq6758 39565853 PMC11639200

[25] X. Wang, W. Pi, S. Hu, H.F. Bao, N. Yao et al., Boosting oxygen evolution reaction performance on NiFe-based catalysts through d-orbital hybridization. Nano-Micro Lett. **17**, 11 (2025). 10.1007/s40820-024-01528-9 PMC11427650 39325091

[26] Q.P. Huang, G.J. Xia, B. Huang, D.L. Xie, J. Wang et al., Activating lattice oxygen by a defect-engineered Fe_2_O_3_-CeO_2_ nano-heterojunction for efficient electrochemical water oxidation. Energy Environ. Sci. **17**(14), 5260–5272 (2024). 10.1039/d4ee01588f

[27] P.C. Ye, K.Q. Fang, H.Y. Wang, Y.H. Wang, H. Huang et al., Lattice oxygen activation and local electric field enhancement by co-doping Fe and F in CoO nanoneedle arrays for industrial electrocatalytic water oxidation. Nat. Commun. **15**, 1012 (2024). 10.1038/s41467-024-45320-0 38307871 PMC10837452

[28] T. Zhang, H.F. Zhao, Z.J. Chen, Q. Yang, N. Gao et al., High-entropy alloy enables multi-path electron synergism and lattice oxygen activation for enhanced oxygen evolution activity. Nat. Commun. **16**, 3327 (2025). 10.1038/s41467-025-58648-y 40199911 PMC11978795

[29] K.H. Miao, W.D. Jiang, Z.Q. Chen, Y. Luo, D. Xiang et al., Hollow-structured and polyhedron-shaped high entropy oxide toward highly active and robust oxygen evolution reaction in a full pH range. Adv. Mater. **36**(8), 2308490 (2024). 10.1002/adma.202308490 38049153

[30] X.M. Li, Z.K. Xie, S. Roy, L.Q. Gao, J. Liu et al., Amorphous high-entropy phosphide nanosheets with multi-atom catalytic sites for efficient oxygen evolution. Adv. Mater. **37**(10), 2410295 (2025). 10.1002/adma.202410295 39713949

[31] H.J.W. Li, Y. Lin, J.Y. Duan, Q.L. Wen, Y.W. Liu et al., Stability of electrocatalytic OER: from principle to application. Chem. Soc. Rev. **53**(21), 10709–10740 (2024). 10.1039/d3cs00010a 39291819

[32] H. Shi, Y.T. Zhou, R.Q. Yao, W.B. Wan, X. Ge et al., Spontaneously separated intermetallic Co_3_Mo from nanoporous copper as versatile electrocatalysts for highly efficient water splitting. Nat. Commun. **11**, 2940 (2020). 10.1038/s41467-020-16769-6 32522988 PMC7287083

[33] H.F. Zhao, L. Li, T. Zhang, J.Q. Yao, X. Peng et al., Discovering high-entropy electrocatalysts through a batch-alloy targeting approach. Sci. Adv. **11**(28), eadx6121 (2025). 10.1126/sciadv.adx6121 40644529 PMC12248279

[34] J. Erlebacher, M.J. Aziz, A. Karma, N. Dimitrov, K. Sieradzki, Evolution of nanoporosity in dealloying. Nature **410**, 450–453 (2001). 10.1038/35068529 11260708

[35] R.Q. Yao, Y.T. Zhou, H. Shi, W.B. Wan, Q.H. Zhang et al., Nanoporous surface high-entropy alloys as highly efficient multisite electrocatalysts for nonacidic hydrogen evolution reaction. Adv. Funct. Mater. **31**(10), 2009613 (2020). 10.1002/adfm.202009613

[36] H. Shi, T.Y. Dai, X.Y. Sun, Z.L. Zhou, S.P. Zeng et al., Dual-intermetallic heterostructure on hierarchical nanoporous metal for highly efficient alkaline hydrogen electrocatalysis. Adv. Mater. **36**(38), 2406711 (2024). 10.1002/adma.202406711 39046064

[37] W. Huang, Y.A. Chang, A thermodynamic analysis of the Ni Al system. Intermetallics **6**(6), 487–498 (1998). 10.1016/s0966-9795(97)00099-x

[38] H. Shi, T.Y. Dai, X.Y. Sun, Z.L. Zhou, Y. Wang et al., High-entropy alloy/intermetallic compound heterostructures for efficient hydrazine oxidation-assisted hydrogen production. Adv. Mater. **37**(45), e12081 (2025). 10.1002/adma.202512081 40852841

[39] H. Shi, X.Y. Sun, Y. Liu, S.P. Zeng, Q.H. Zhang et al., Multicomponent intermetallic nanoparticles on hierarchical metal network as versatile electrocatalysts for highly efficient water splitting. Adv. Funct. Mater. **33**(19), 2214412 (2023). 10.1002/adfm.202214412

[40] W.L. Yu, Z. Chen, Y.L. Fu, W.P. Xiao, B. Dong et al., Superb all-pH hydrogen evolution performances powered by ultralow Pt-decorated hierarchical Ni-Mo porous microcolumns. Adv. Funct. Mater. **33**(4), 2210855 (2023). 10.1002/adfm.202210855

[41] Z.X. Zhu, L. Luo, Y.X. He, M. Mushtaq, J.Q. Li et al., High-performance alkaline freshwater and seawater hydrogen catalysis by sword-head structured Mo_2_N-Ni_3_Mo_3_N tunable interstitial compound electrocatalysts. Adv. Funct. Mater. **34**(8), 2306061 (2024). 10.1002/adfm.202306061

[42] Y. Yao, Q. Dong, A. Brozena, J. Luo, J. Miao et al., High-entropy nanoparticles: synthesis-structure-property relationships and data-driven discovery. Science **376**(6589), 3103–3114 (2022). 10.1126/science.abn3103 35389801

[43] W.L. Hsu, C.W. Tsai, A.C. Yeh, J.W. Yeh, Clarifying the four core effects of high-entropy materials. Nat. Rev. Chem. **8**, 471–485 (2024). 10.1038/s41570-024-00602-5 38698142

[44] N. Kar, S.E. Skrabalak, Synthetic methods for high-entropy nanomaterials. Nat. Rev. Mater. **10**, 638–653 (2025). 10.1038/s41578-025-00829-8

[45] D.X. Li, C. Liu, S.S. Tao, J.M. Cai, B. Zhong et al., High-entropy electrode materials: synthesis, properties and outlook. Nano-Micro Lett. **17**, 22 (2025). 10.1007/s40820-024-01504-3 PMC11436529 39331215

[46] G.L. Cao, S. Yang, J.C. Ren, W. Liu, Electronic descriptors for designing high-entropy alloy electrocatalysts by leveraging local chemical environments. Nat. Commun. **16**, 1251 (2025). 10.1038/s41467-025-56421-9 39893203 PMC11787369

[47] R.Q. Yao, H. Shi, W.B. Wan, Z. Wen, X.Y. Lang et al., Flexible Co-Mo-N/Au electrodes with a hierarchical nanoporous architecture as highly efficient electrocatalysts for oxygen evolution reaction. Adv. Mater. **32**(10), 1907214 (2020). 10.1002/adma.201907214 31999014

[48] G.H. Wang, Z.J. Chen, J.L. Zhu, J.Z. Xie, W. Wei et al., High-entropy amorphous catalysts for water electrolysis: a new frontier. Nano-Micro Lett. **18**, 77 (2026). 10.1007/s40820-025-01936-5 PMC12535958 41111133

[49] H.S. Hu, X.L. Wang, J.P. Attfield, M.H. Yang, Metal nitrides for seawater electrolysis. Chem. Soc. Rev. **53**(1), 163–203 (2024). 10.1039/d3cs00717k 38019124

[50] L. An, J.Y. Li, Y.M. Sun, J.M. Zhu, J.Z.Y. Seow et al., Deciphering water oxidation catalysts: the dominant role of surface chemistry over reconstruction degree in activity promotion. Nano-Micro Lett. **17**, 70 (2024). 10.1007/s40820-024-01424-2 PMC11599692 39589691

[51] F.Y. Wu, F.Y. Tian, M.G. Li, S. Geng, L.Y. Qiu et al., Engineering lattice oxygen regeneration of NiFe layered double hydroxide enhances oxygen evolution catalysis durability. Angew. Chem. Int. Ed. **64**(1), e202413250 (2025). 10.1002/anie.202413250 39451124

[52] Z.W. Cai, J. Liang, Z.X. Li, T.Y. Yan, C.X. Yang et al., Stabilizing NiFe sites by high-dispersity of nanosized and anionic Cr species toward durable seawater oxidation. Nat. Commun. **15**, 6624 (2024). 10.1038/s41467-024-51130-1 39103352 PMC11300586

[53] L. Shao, X.D. Han, L. Shi, T.Z. Wang, Y.S. Zhang et al., In situ generation of molybdate-modulated nickel-iron oxide electrodes with high corrosion resistance for efficient seawater electrolysis. Adv. Energy Mater. **14**(4), 2303261 (2024). 10.1002/aenm.202303261

[54] S.P. Zeng, H. Shi, T.Y. Dai, Y. Liu, Z. Wen et al., Lamella-heterostructured nanoporous bimetallic iron-cobalt alloy/oxyhydroxide and cerium oxynitride electrodes as stable catalysts for oxygen evolution. Nat. Commun. **14**, 1811 (2023). 10.1038/s41467-023-37597-4 37002220 PMC10066221

[55] K.H. Yue, R.H. Lu, M.B. Gao, F. Song, Y. Dai et al., Polyoxometalated metal-organic framework superstructure for stable water oxidation. Science **388**(6745), 430–436 (2025). 10.1126/science.ads1466 40273253

[56] S.J. Liu, Z.G. Zhang, K. Dastafkan, Y. Shen, C. Zhao et al., Yttrium-doped NiMo-MoO_2_ heterostructure electrocatalysts for hydrogen production from alkaline seawater. Nat. Commun. **16**, 773 (2025). 10.1038/s41467-025-55856-4 39824853 PMC11742019

[57] L. Sun, J.A. Yuwono, S.L. Zhang, B. Chen, G.J. Li et al., High entropy alloys enable durable and efficient lithium-mediated CO_2_ redox reactions. Adv. Mater. **36**(25), 2401288 (2024). 10.1002/adma.202401288 38558119

[58] F.R. Qian, L.S. Peng, D.F. Cao, W. Jiang, C.S. Hu et al., Asymmetric active sites originate from high-entropy metal selenides by joule heating to boost electrocatalytic water oxidation. Joule **8**(8), 2342–2356 (2024). 10.1016/j.joule.2024.06.004

[59] J.Z. Huang, A.H. Clark, N. Hales, K. Crossley, J. Guehl et al., Oxidation of interfacial cobalt controls the pH dependence of the oxygen evolution reaction. Nat. Chem. **17**, 856 (2025). 10.1038/s41557-025-01784-1 40155757 PMC12141032

[60] X.G. Sun, W. Shen, H. Liu, P.X. Xi, M. Jaroniec et al., Corrosion-resistant NiFe anode towards kilowatt-scale alkaline seawater electrolysis. Nat. Commun. **15**, 10351 (2024). 10.1038/s41467-024-54754-5 39609468 PMC11605038

