# Supplementary material for: Multicomponent Nitride/High-Entropy Alloy Heterostructures as Highly Active and Stable Electrocatalysts for Ampere-Level Water Oxidation
Source: Nanomicro Lett. 2026 Sep 11;19:46. doi: 10.1007/s40820-026-02316-3 (PMC13562473; doi:10.1007/s40820-026-02316-3)
Supplement: Supplementary file 1 [file 40820_2026_2316_MOESM1_ESM.docx]

Supporting Information for

**Multicomponent Nitride/High-Entropy Alloy Heterostructures as Highly Active and Stable Electrocatalysts for Ampere-Level Water Oxidation**

Xin-Ying Sun^1✝^, Tian-Yi Dai^1✝^, Rui-Qi Yao^2^, Yun-Fei Chang^1^, Zhi-Lan Zhou^1^, Ying Wang^1^, Tong-Hui Wang^1^, Gao-Feng Han^1^, Zi Wen^1^, Hang Shi^1^*, Xing-You Lang^1^*, Qing Jiang^1^*

^1^ Key Laboratory of Automobile Materials (Jilin University), Ministry of Education, School of Materials Science and Engineering, Jilin University, Changchun 130022, P. R. China

^2^ Key Laboratory of Polyoxometalate and Reticular Material Chemistry of Ministry of Education, Faculty of Chemistry, Northeast Normal University, Changchun 130024, P. R. China

^✝^Xin-Ying Sun and Tian-Yi Dai contributed equally to this work.

* Corresponding authors. E-mail: [xylang@jlu.edu.cn](mailto:xylang@jlu.edu.cn) (Xing-You Lang); [shihang@jlu.edu.cn](mailto:shihang@jlu.edu.cn) (Hang Shi); [jiangq@jlu.edu.cn](mailto:jiangq@jlu.edu.cn) (Qing Jiang)

**S1 Supplementary Figures**

**Fig. S1** EDS elemental characterizations of precursor alloys. EDS spectra of precursor Ni_13_Fe_2_Co_2_Mo_6_Cr_2_Al_75_ (**a**), Ni_21_Fe_2_Co_2_Al_75_ (**b**), Ni_19_Mo_6_Al_75_ (**c**) and Ni_25_Al_75_ (**d**) alloys.

**Fig. S2** XRD characterizations of precursor alloys. XRD patterns of precursor Ni_13_Fe_2_Co_2_Mo_6_Cr_2_Al_75_, Ni_21_Fe_2_Co_2_Al_75_, Ni_19_Mo_6_Al_75_ and Ni_25_Al_75_ alloys. The line patterns show reference cards 04-0787 for α-Al (dark cyan line), 97-005-8040 for Al_3_Ni (navy line), 29-0042 for Al_13_Fe_4_ (lilac line), 06-0699 for Al_9_Co_2_ (wine line), 48-1564 for Al_84.6_Cr_15.4_ (purple line), 65-7072 for Al_4_Mo (light red line), and 65-5480 for MoNi_4_ (dark yellow line) according to JCPDS.

**Fig. S3** (**a**) SEM image of nanoporous (MoCr)(NiFeCo)_4_/HEA electrode, which is fabricated by chemically dealloying Ni_13_Fe_2_Co_2_Cr_2_Mo_6_Al_75_ precursor alloy. (**b**) XRD patterns of nanoporous (MoCr)(NiFeCo)_4_/HEA, MoNi_4_/Ni, and Ni electrodes. The line patterns show reference cards 65-5480 and 04-0850 for MoNi_4_ and Ni according to JCPDS, respectively.

**Fig. S4** (**a**) SEM images of nanoporous (NiFeCo)_2_(MoCr)_3_O_8_/HEA electrode. (**b**) XRD pattern of (NiFeCo)_2_(MoCr)_3_O_8_/HEA electrode. The line patterns show reference cards 37-0855 and 04-0850 for Ni_2_Mo_3_O_8_ and Ni according to JCPDS, respectively.

**Fig. S5** XPS survey spectra of nanoporous (NiFeCo)_3_(MoCr)_3_N/HEA electrode.

**Fig. S6** EDS elemental characterizations of nanoporous hybrid electrodes. EDS spectra of nanoporous (NiFeCo)_3_(MoCr)_3_N/HEA (**a**), N-NiFeCo/NiFeCo (**b**), Ni_3_Mo_3_N/Ni (**c**) and N-Ni/Ni (**d**) electrodes.

**Fig. S7** Morphology characterization of electrodes. Typical SEM images of nanoporous N-NiFeCo/NiFeCo (**a**), Ni_3_Mo_3_N/Ni (**b**) and N-Ni/Ni (**c**) electrodes.

**Fig. S8** SEM image (**a**), HRTEM image (**b**), XRD pattern (**c**), STEM and the corresponding EDS elemental mappings of Ni, Fe, Co, Cr, Mo, Al, N and O (**d**) for phase-reconstructed nanoporous (NiFeCo)_3_(MoCr)_3_N/HEA electrode, i.e., nanoporous NiFeCoMoCr.

**Fig. S9** EDS spectra of phase-reconstructed (NiFeCo)_3_(MoCr)_3_N/HEA electrode.

**Fig. S10** The concentration of Cr ions in the KOH electrolyte as a function of OER proceeding time. Here the Cr ions result from the dissolution of Cr from nanoporous (NiFeCo)_3_(MoCr)_3_N/HEA electrode during the OER.

**Fig. S11** In-situ Raman spectra of nanoporous (NiFeCo)_3_(MoCr)_3_N/HEA electrode at various applied potentials in 1 M KOH.

**Fig. S12** Raman spectra of NiFeCoMoCrOOH-N/HEA and NiFeCoMoCrOOH/HEA.

**Fig. S13** High-resolution Ni 2*p*, Co 2*p*, Fe 2*p*, Cr 2*p*, O 1*s*, N 1*s* and Mo 3*d* XPS spectra of nanoporous (NiFeCo)_3_(MoCr)_3_N/HEA.

**Fig. S14** Comparisons of high-resolution Ni 2*p*_3/2_, Mo 3*d*_5/2_ and O 1*s* XPS spectra of NiFeCoMoCrOOH-N/HEA and NiMoOOH-N/Ni.

**Fig. S15** (**a**) Ni K-edge XANES spectra of NiFeCoMoCrOOH-N/HEA, NiFeCoMoCrOOH/HEA and (NiFeCo)_3_(MoCr)_3_N/HEA. (**b**) Mo K-edge XANES spectra of NiFeCoMoCrOOH-N/HEA, NiFeCoMoCrOOH/HEA and (NiFeCo)_3_(MoCr)_3_N/HEA. (**c**) Fe K-edge XANES spectra of NiFeCoMoCrOOH-N/HEA, (NiFeCo)_3_(MoCr)_3_N/HEA and Fe_3_O_4_. (**d**) Co K-edge XANES spectra of NiFeCoMoCrOOH-N/HEA, (NiFeCo)_3_(MoCr)_3_N/HEA and CoO. (e) Cr K-edge XANES spectra of NiFeCoMoCrOOH-N/HEA, (NiFeCo)_3_(MoCr)_3_N/HEA and Cr_2_O_3_.

**Fig. S16** (**a-b**) Fourier-transformed R-space Ni K-edge (**a**) and Mo K-edge (**b**) EXAFS spectra of nanoporous (NiFeCo)_3_(MoCr)_3_N/HEA, (NiFeCo)_2_(MoCr)_3_O_8_/HEA, Ni_3_Mo_3_N/Ni after phase reconstruction, comparing with the pristine (NiFeCo)_3_(MoCr)_3_N/HEA and Ni_3_Mo_3_N/Ni electrodes. (**c**) Fourier-transformed R-space Fe K-edge EXAFS spectra of NiFeCoMoCrOOH-N/HEA, (NiFeCo)_3_(MoCr)_3_N/HEA and Fe_3_O_4_. (**d**) Fourier-transformed R-space Co K-edge EXAFS spectra of NiFeCoMoCrOOH-N/HEA, (NiFeCo)_3_(MoCr)_3_N/HEA and CoO. (**e**) Fourier-transformed R-space Cr K-edge EXAFS spectra of nanoporous (NiFeCo)_3_(MoCr)_3_N/HEA and (NiFeCo)_2_(MoCr)_3_O_8_/HEA after phase reconstruction, comparing with the pristine (NiFeCo)_3_(MoCr)_3_N/HEA electrode.

**Fig. S17** Current-potential curve of Pt wire used as the working electrode in H_2_-saturated 1 M KOH electrolyte with the scan rate of 1 mV s^−1^ to calibrate the Ag/AgCl electrode with respect to RHE. *E*_RHE_ = *E*_Ag/AgCl_ + 1.016 V.

**Fig. S18** Comparison of charge transfer resistance (*R*_CT_) for nanoporous (NiFeCo)_3_(MoCr)_3_N/HEA, Ni_3_Mo_3_N/Ni, N-NiFeCo/NiFeCo, N-Ni/Ni, RuO_2_/Ni and (NiFeCo)_2_(MoCr)_3_O_8_/HEA electrodes according to EIS analysis for OER.

**Fig. S19** OER polarization curves of nanoporous (NiFeCo)_3_(MoCr)_3_N/HEA electrodes with 0% to 100% iR compensation in 1 M KOH electrolyte. Scan rate: 1 mV s^−1^.

**Fig. S20** (**a**) EIS spectra for eight nanoporous (NiFeCo)_3_(MoCr)_3_N/HEA electrodes, which are prepared by the same alloying/dealloying and annealing procedures. (**b**) Series resistance (*R*_S_) of eight nanoporous (NiFeCo)_3_(MoCr)_3_N/HEA electrodes.

**Fig. S21** Electrochemical double-layer capacitance measurements. (**a-i**) CV curves in the potential windows ranging from −0.85 V to −0.00 V (vs. Ag/AgCl) for reconstructed (NiFeCo)_3_(MoCr)_3_N/HEA (**a**), (NiFeCo)_2_(MoCr)_3_O_8_/HEA (**b**), (NiFe)_3_(MoCr)_3_N/HEA (**c**), (NiFeCo)_3_Mo_3_N/HEA (**d**), (NiCo)_3_(MoCr)_3_N/HEA (**e**), Ni_3_Mo_3_N/Ni (**f**), RuO_2_/Ni (**g**), N-NiFeCo/NiFeCo (**h**), N-Ni/Ni (**i**) electrodes at different scan rates in 1 M KOH. (**j**, **k**) Double-layer capacitances (C_dl_) calculated by the differences of current densities at −0.05 V (vs. Ag/AgCl) for the investigated electrodes as a linear function of scan rates.

**Fig. S22** EDS elemental characterizations of precursor alloys. EDS spectra of precursor Ni_15_Fe_2_Co_2_Mo_6_Al_75_ (**a**), Ni_15_Fe_2_Cr_2_Mo_6_Al_75_ (**b**) and Ni_19_Co_2_Cr_2_Mo_6_Al_75_ (**c**) alloys.

**Fig. S23** XRD characterizations of precursor alloys. XRD patterns of precursor Ni_15_Fe_2_Mo_6_Cr_2_Al_75_, Ni_15_Fe_2_Co_2_Mo_6_Al_75_ and Ni_15_Co_2_Mo_6_Cr_2_Al_75_. The line patterns show reference cards 04-0787 for α-Al (dark cyan line), 97-005-8040 for Al_3_Ni (navy line), 29-0042 for Al_13_Fe_4_ (lilac line), 06-0699 for Al_9_Co_2_ (wine line), 48-1564 for Al_84.6_Cr_15.4_ (purple line), 65-7072 for Al_4_Mo (light red line), and 65-5480 for MoNi_4_ (dark yellow line) according to JCPDS.

**Fig. S24** EDS elemental characterizations of nanoporous hybrid electrodes. EDS spectra of nanoporous (NiFeCo)_3_Mo_3_N/HEA (**a**), (NiFe)_3_(MoCr)_3_N/HEA (**b**) and (NiCo)_3_(MoCr)_3_N/HEA (**c**) electrodes.

**Fig. S25** XRD patterns of (NiFeCo)_3_Mo_3_N/HEA, (NiFe)_3_(MoCr)_3_N/HEA and (NiCo)_3_(MoCr)_3_N/HEA electrodes. The line patterns show reference cards 49-1336 and 04-0850 for Ni_3_Mo_3_N and Ni according to JCPDS, respectively.

**Fig. S26** Morphology characterization of electrodes. Typical SEM images of (NiFe)_3_(MoCr)_3_N/HEA (**a**), (NiFeCo)_3_Mo_3_N/HEA (**b**) and (NiCo)_3_(MoCr)_3_N/HEA (**c**) electrodes.

**Fig. S27** (**a**) OER polarization curves of nanoporous (NiFeCo)_3_(MoCr)_3_N/HEA, (NiFeCo)_3_Mo_3_N/HEA, (NiFe)_3_(MoCr)_3_N/HEA and (NiCo)_3_(MoCr)_3_N/HEA electrodes in 1 M KOH electrolyte. Scan rate: 1 mV s^−1^. (**b**) Tafel slopes for different electrodes according to the OER polarization curves in panel (**a**).

**Fig. S28** (**a**, **b**) Ion Chromatograph (IC) of various concentrations of MoO_4_^2–^ (**a**) and CrO_4_^2–^ (**b**). (**c**, **d**) Calibration curves for MoO_4_^2–^ (**c**) and CrO_4_^2–^ (**d**) via IC measurement. (**e**) The IC spectra of MoO_4_^2–^ and CrO_4_^2–^adsorbed on the nanoporous (NiFeCo)_3_(MoCr)_3_N/HEA electrode. (**f**) The concentrations of adsorbed MoO_4_^2–^ and CrO_4_^2–^ normalized by electrode area.

**Fig. S29** OER polarization curves of nanoporous (NiFeCo)_3_(MoCr)_3_N/HEA with and without MoO_4_^2−^ and CrO_4_^2−^ [(MoCr)O_4_^2−^] anions. Electrolyte: 1 M KOH.

**Fig. S30** OER polarization curves of nanoporous N-NiFeCo/NiFeCo electrodes in 1 M KOH electrolyte with and without 5 mM MoO_4_^2−^ and/or 5 mM CrO_4_^2−^ anions.

**Fig. S31** **Reproducibility of nanoporous (NiFeCo)_3_(MoCr)_3_N/HEA electrode in 1 M KOH electrolyte.** (**a**) The OER polarization curves for eight nanoporous (NiFeCo)_3_(MoCr)_3_N/HEA electrodes, which are prepared by the same alloying/dealloying and annealing procedures. (**b**) Overpotentials at different current densities of 1, 2 and 3 A cm^−2^ for eight (NiFeCo)_3_(MoCr)_3_N/HEA electrodes from panel a.

**Fig. S32** Typical surface (**a**) and cross-sectional (**b**) SEM images of the (NiFeCo)_3_(MoCr)_3_N/HEA electrode after OER stability measurement.

**Fig. S33** High-resolution Ni 2*p* (**a**), Co 2*p* (**b**), Fe 2*p* (**c**), Cr 2 (**d**), Mo 3*d* (**e**), O 1*s* (**f**) and N 1*s* (**g**) XPS spectra of (NiFeCo)_3_(MoCr)_3_N/HEA after OER stability measurement.

**Fig S34** Atomic structures of NiFeCoCrMoOOH (**a**, **b**), NiFeCoCrMoOOH-N (**c**, **d**), and NiFeCoCrMoOOH-N with CrO_4_^2−^ (**e**, **f**) for DFT calculations.

**Fig. S35** Gibbs free energy diagram of OER on the Ni, Fe and O sites of NiFeCoMoCrOOH.

**Fig. S36** The PDOS of *p*-orbital in *OH and *d*-orbital in Fe of NiFeCoMoCrOOH-N. Inset: The differential charge density distribution of *OH adsorbed on Fe site of NiFeCoMoCrOOH-N, where pink and yellow isosurfaces denote electron density accumulation and depletion, respectively. The isosurface level is 0.002 |e| Å^−3^.

**Fig. S37** (**a**) Gibbs free energy diagram of OER on NiFeCoMoCrOOH and NiFeCoMoCrOOH-N following AEM path. (**b**) Interface configuration of NiFeCoMoCrOOH-N at four different stages via AEM path.

**Fig. S38** (**a**, **b**) OER polarization curves of nanoporous (NiFeCo)_3_(MoCr)_3_N/HEA (**a**) and N-NiFeCo/NiFeCo (**b**) electrodes in KOH electrolytes with different pH. (**c**) The pH dependence of OER current densities at the overpotential of 300 mV for nanoporous (NiFeCo)_3_(MoCr)_3_N/HEA and N-NiFeCo/NiFeCo electrodes.

**Fig. S39** Alkaline HER electrocatalytic properties. (**a**) Representative HER polarization curves for (MoCr)(NiFeCo)_4_/HEA, MoNi_4_/Ni, Pt/C/Ni and Ni electrodes. Scan rate: 1 mV s^−1^. Electrolyte: 1 M KOH. (**b**) Tafel plots for (MoCr)(NiFeCo)_4_/HEA, MoNi_4_/Ni, Pt/C/Ni and Ni electrodes based on the polarization curves in (**a**).

**Fig. S40** Durability of two (NiFeCo)_3_(MoCr)_3_N/HEA||(MoCr)(NiFeCo)_4_/HEA electrolysers at the input voltage of 1.92 V.

**S2 Supplementary Notes**

**Note S1 Calculation of specific current density (*j*_s_)**

*j*_s_ = *j*_geo_/*A*_ECSA_ (S1)

*A*_ECSA_ = C_dl_/C_s_	(S2)

Herein, *j*_geo_ and *j*_s_ refer to the current per geometric area and electrochemical specific area, respectively. *A*_ECSA_ is electrochemical specific area per geometric area and C_s_ is the specific capacitance.

At the overpotential of 300 mV,

*j*_s, (NiFeCo)3(MoCr)3N/HEA_ = 0.404 mA cm^–2^, *j*_s, (NiFeCo)2(MoCr)3O8/HEA_ = 0.009 mA cm^–2^,

*j*_s, (NiFeCo)3Mo3N/HEA_ = 0.155 mA cm^–2^, *j*_s, (NiFe)3(MoCr)3N/HEA_ = 0.320 mA cm^–2^,

*j*_s, (NiCo)3(MoCr)3N/HEA_ = 0.007 mA cm^–2^, *j*_s, Ni3Mo3N/Ni_ = 0.011 mA cm^–2^,

*j*_s, N-NiFeCo/NiFeCo_ = 0.009 mA cm^–2^, *j*_s, N-Ni/Ni_ = 0.005 mA cm^–2^,

*j*_s, RuO2/Ni_ = 0.023 mA cm^–2^.

**Note S2 Measurement of adsorbed MoO_4_^2−^ and CrO_4_^2−^**

The concentrations of MoO_4_^2−^ and CrO_4_^2−^ around nanoporous (NiFeCo)_3_(MoCr)_3_N/HEA electrode was measured by ion chromatograph. Briefly, the activated nanoporous (NiFeCo)_3_(MoCr)_3_N/HEA electrode was operated at the overpotential of 300 mV for 180 s in 1 M KOH electrolyte, and then immersed in 10 mL ultrapure water with shaking for 60 s to completely release surface-adsorbed MoO_4_^2−^ and CrO_4_^2−^. After repeating the above process ten times, the collected solution was ultrasonicated for 600 s to ensure homogeneous dispersion and subsequently directly analyzed via ion chromatography.

**Note S3 Calculation of TOF values**

TOF = *N*_TH_/*N*_AS_	(S3)

Herein, *N*_TH_ and *N*_AS_ correspond to the number of total oxygen turnovers per geometric area and the number of active sites per geometric area, respectively. The values of *N*_TH_ and *N*_AS_ are calculated according to the equations:

*N*_TH_ = ($\text{j}\frac{\text{mA}}{\text{cm²}}$)($\frac{\text{1 Cs}^{\text{­1}}}{\text{1000 mA}}$)($\frac{{\text{1}\text{ mol e}}^{\text{­}}}{\text{96485.3 C}}$)($\frac{\text{1 mol }\text{O}_{\text{2}}}{\text{4}\text{ mol }\text{e}^{\text{­}}}$)($\frac{\text{6.022}\text{*}\text{10}^{\text{23}}\text{ }\text{O}_{\text{2}}\text{ molecules}}{\text{1 mol }\text{O}_{\text{2}}}$)	(S4)

*N*_AS_ = ${\text{(}\frac{\text{Number of atoms/unit cell}}{\text{Volume/unit cell}}\text{)}}^{\frac{\text{2}}{\text{3}}}$	(S5)

Herein, the ideal NiOOH model is adopted to replace N-doped NiFeCoCrMoOOH for subsequent computational estimation. For the Hexagonal NiOOH with *a* = *b* = 2.82 Å and *c* = 20.57 Å, the volume of unit cell containing 4 atoms is 141.5 Å^3^. Therefore, the *N*_AS_ = 0.928 × 10^15^ atoms cm^−2^.

At the overpotential of 300 mV,

TOF_(NiFeCo)3(MoCr)3N/HEA_ = 0.680 s^−1^.

**Note S4 The reaction steps of the CrO_4_^2−^-mediated AAM pathway**

OH^−^ + * → *OH + e^−^	(S6)

*OH + OH^−^ + CrO_4_^2−^ → *OOH + HCrO_4_^−^ + 2e^−^	(S7)

*OOH + OH^−^ → * + O_2_ + H_2_O + e^−^	(S8)

HCrO_4_^−^ + OH^−^ → CrO_4_^2−^ + H_2_O	(S9)

**The Gibbs free energy change for each step in the CrO_4_^2−^-mediated AAM pathway**

∆*G*_1_ = *G*_*OH_ − *G*_OH−_ − *G*_*_ − e*U*	(S10)

∆*G*_2_ = *G*_*OOH_*_+_*_*H_ − *G*_*OH_ − *G*_OH−_ − e*U*	(S11)

∆*G*_3_ = *G*_*H_ *+ G*_O2_ *+ G*_H+_ − *G*_*OOH_*_+_*_*H_ − e*U*	(S12)

∆*G*_4_ = *G*_*_ *+ G*_H+_ − *G*_*H_ − e*U*	(S13)

where e was the energy of the electron and *U* was the electrode potential versus RHE.

**Note S5 Calculation of energy efficiency**

Energy efficiency (%) = $\frac{E_{\mathrm{output}}}{E_{\mathrm{input}}}$ = $\frac{M_{H2} \times\mathrm{HHV}_{H2} (kJ g^{-1}) \times1000}{I (A) \times U (V) \times t (s)}$	(S14)

Here, *M*_H2_ is the mass of hydrogen generated over a specific time interval, HHV_H2_ is the higher heating value of hydrogen (141.7 kJ g_H2_^−1^), *I* is the current, *U* is the cell voltage, *t* is the time.

**Note S6 Calculation of hydrogen production cost**

The hydrogen production cost is calculated based on an ideal 1 MW alkaline water electrolysis system. The annual expenditure of the system consists of capital expenditure (CAPEX) and operational expenditure (OPEX), with CAPEX further divided into the cost of the electrolyzer stack and balance of plant (BOP). Assuming the plant has a daily hydrogen production capacity of 330 kg and the electrolyzer stack and BOP have an expected service life of 10 years with 350 operating days per year. The price of a typical alkaline water electrolyzer stack is 1070.6 USD, and the cost for electrode and diaphragm replacement is 171.3 USD. The BOP accounts for 58% of CAPEX, with an annual maintenance cost at 2.5% of CAPEX and a discount rate of 10% [S1, S2]. The annual labor cost for plant operation equals 0.3% of CAPEX. The base electricity price and renewable energy electricity price are 0.05 USD kWh^–1^ and 0.02 USD kWh^–1^ respectively [S3]. Based on the above assumptions, the production cost of hydrogen is calculated according to the following equations:

Total current (A) = $\frac{330 ({kg day}^{-1}) \times2 (e^{-}) \times96485 ({C mol}^{-1})}{2 ({kg mol}^{-1}) \times24 ({hour day}^{-1}) \times3600 ({s hour}^{-1}) \times Energy efficiency (\%)}$

(S15)

Power consumed (kW) = $\frac{Total current (A) \times Electrolyzer stack voltage (V)}{1000}$

(S16)

Electricity cost (USD year^–1^) = Power consumed (kW) × 24 (hour day^–1^) × 350 (day year^–1^) × Electricity price (USD kWh^–1^)	(S17)

Total stack area required (m^2^) = $\frac{Total current (A)}{Current density (A cm^{-2}) \times10000}$	(S18)

Stack cost (USD) = Total stack area required (m^2^) × Stack price (USD m^–2^)

(S19)

BOP cost (USD) = $\frac{\text{Stack cost (USD) × 58\%}}{\text{1 }\text{–}\text{ 58\%}}$ (S20)

Capital recovery factor = $\frac{Discount rate \times{(1 + Discount rate)}^{Plant lifetime}}{{(1 + Discount rate)}^{Plant lifetime}-1}$	(S21)

Annual CAPEX (USD year^–1^) = (Stack cost + BOP cost) × Capital recovery factor

(S22)

Maintenance cost (USD year^–1^) = CAPEX (USD) × 2.5%	(S23)

Labor cost (USD year^–1^) = CAPEX (USD) × 0.3%	(S24)

Electrode/diaphragm replacement cost (USD year^–1^) = $\frac{Electrode/diaphragm total price (USD m^{-2} ) \times Total stack area required (m^{-2})}{Electrode/diaphragm lifetime (year)}$

(S25)

Water cost (USD year^−1^) = 330 (kg day^−1^) × 350 (day year^−1^) × 45 (kg_water_ kg_H2_^−1^) × DI water price (USD kg^−1^)	(S26)

Annual OPEX (USD year^−1^) = Electricity cost + (Maintenance cost + Electrode/diaphragm replacement cost) + Labor cost + Water cost	(S27)

Hydrogen levelized production cost (USD kg^−1^) = $\frac{Annual CAPEX (USD \mathrm{year}^{-1}) + Annual OPEX (USD \mathrm{year}^{-1})}{350 (day \mathrm{year}^{-1}) \times330 (kg \mathrm{day}^{-1})}$	(S28)

**S3 Supplementary Tables**

**Table S1.** Comparisons of current density at the overpotential of 300 mV, Tafel slope and long-term stability for (NiFeCo)_3_(MoCr)_3_N/HEA electrode with representative electrocatalysts reported previously

| **Electrocatalysts** | **Current**  **density**  **(mA cm^–2^)** | **Tafel slope**  **(mV dec^–1^)** | **Stability** | | **Refs.** |
| --- | --- | --- | --- | --- | --- |
|  |  |  | **Current density**  **(mA cm^–2^)** | **Time**  **(h)** |  |
| (NiFeCo)_3_(MoCr)_3_N/HEA | 2466 | 30 | 1000 | 1600 | This work |
| (NiFeCo)_2_(MoCr)_3_O_8_/HEA | 18 | 295 | 1 | 238 | This work |
| Ni_3_Mo_3_N/Ni | 55 | 53 | 17 | 470 | This work |
| NiFeOOH@SO_4_ | 675 | 56 | 500 | 300 | [S4] |
| FeNiHOF | 1900 | 35 | 1000 | 1000 | [S5] |
| Fe, F-CoO | 675 | 42 | 500 | 300 | [S6] |
| NiMoN@NiFeN | 198 | 59 | 100 | 48 | [S7] |
| FeCoNiZnMo | 282 | 49 | 500 | 800 | [S8] |
| FeOOH/Co(OH)_2_ | 817 | 38 | 100 | 150 | [S9] |
| FeZnNNiCoV | 709 | 36 | 1000 | 1030 | [S10] |
| FeCoNiMnCr HEA-HEO/C | 95 | 42 | 100 | 100 | [S11] |
| Co_2_Fe_0.25_V_0.75_O_4_ | 331 | 41 | 500 | 600 | [S12] |
| Bi/BiCeO_1.8_H | 271 | 36 | 200 | 500 | [S13] |
| (FeCoNiCrCu)Se*_x_* | 84 | 56 | 100 | 500 | [S14] |
| (FeCoNi)_2_Nb | 100 | 64 | 500 | 1600 | [S15] |
| F-NiFeO | 1096 | 47 | 1000 | 100 | [S16] |
| Fe_2_O_3_@CeO_2_ | 791 | 58 | 1000 | 100 | [S17] |
| Fe-Ni(P_4_O_11_) | 187 | 43 | 100 | 100 | [S18] |
| CoFeMoO | 168 | 54 | 10 | 1000 | [S19] |
| NiRuCoO*_x_* | 68 | 54 | 10 | 110 | [S20] |
| Ir@FeNiSe_2_ | 911 | 29 | 10 | 50 | [S21] |
| Zn-NiOOH/NF | 450 | 31 | 200 | 800 | [S22] |
| Ir-NiO | 100 | 41 | 20 | 20 | [S23] |

**Table S2** ICP-OES results of the electrolyte after the long-term OER stability test of nanoporous (NiFeCo)_3_(MoCr)_3_N/HEA electrode at the overpotential of 270 mV in 1 M KOH electrolyte for 1600 h

|  | **Concentration (mg/L)** |
| --- | --- |
| Ni | 0.0020 |
| Fe | 0.0075 |
| Co | 0.0020 |
| Cr | 0.0691 |
| Mo | 0.1571 |
| Al | 0.1335 |

**Table S3** Comparisons of the overall splitting water performance of (NiFeCo)_3_(MoCr)_3_N/HEA||(MoCr)(NiFeCo)_4_/HEA electrolyser with representative electrolysers reported previously in 1 M KOH electrolyte

| **Electrocatalysts** | ***η*_100_**  **(V)** | **Stability** | | **Refs.** |
| --- | --- | --- | --- | --- |
|  |  | **Current density**  **(mA cm^–2^)** | **Time**  **(h)** |  |
| (NiFeCo)_3_(MoCr)_3_N/HEA\|\|(MoCr)(NiFeCo)_4_/HEA | 1.43 | 1250 | 1700 | This work |
| FeNiHOF\|\|Ni | 1.71 | 1000 | 500 | [S5] |
| NiMoN@NiFeN\|\|NiMoN | 1.56 | 500 | 100 | [S7] |
| MoNi_4_/MoO_2_\|\|FeOOH/Co(OH)_2_ | 1.54 | 100 | 40 | [S9] |
| FeZnNNiCoV\|\|FeZnNNiCoV | 1.54 | 1000 | 80 | [S10] |
| Bi/BiCeO_1.8_H\|\|Pt/C | 1.57 | 1000 | 100 | [S13] |
| (FeCoNi)_2_Nb\|\|(FeCoNi)_2_Nb | 1.56 | 500 | 140 | [S15] |
| Fe_2_O_3_@CeO_2_\|\|Pt/C | 1.60 | 1000 | 100 | [S17] |
| NiRuCoO*_x_*\|\|Pt/C | 1.66 | 500 | 1500 | [S20] |
| NiO/Mo_2_C\|\|NiO/Mo_2_C | 1.86 | 10 | 120 | [S21] |
| NiFeMo/NF\|\|NiFeMo/NF | 1.66 | 500 | 600 | [S24] |
| Ag/CoV-LDH\|\|Pt/C | 1.55 | 10 | 24 | [S25] |
| P-CoVO@NF\|\|CoN/VN@NF | 1.61 | 500 | 1000 | [S26] |
| Ru-Ni(OH)_2_\|\|Ru-Ni(OH)_2_ | 1.5 | 30 | 65 | [S27] |
| Co_9_S_8_-Fe@C\|\|Co_9_S_8_-Fe@C | 1.82 | 10 | 24 | [S28] |
| Co_0.15_-Ni(NO_3_)_2_OH_2_\|\|Co_0.15_-Ni(NO_3_)_2_OH_2_ | 1.46 | 1000 | 24 | [S29] |
| FeCoNi-C-N\|\|FeCoNi-N-C | 1.60 | 500 | 720 | [S30] |
| NiFe_0.25_Cr_1.75_O_4_\|\|NiFe_0.25_Cr_1.75_O_4_ | 1.45 | 200 | 80 | [S31] |
| WC*_1-x_*/Mo_2_C@C\|\|WC*_1-x_*/Mo_2_C@C | 1.82 | 200 | 100 | [S32] |
| Co_3_Mo_3_N/Co_4_N/Co\|\|Co_3_Mo_3_N/Co_4_N/Co | 1.87 | 200 | 100 | [S33] |
| Ir\|\|Pt-Ir | 1.65 | 500 | 100 | [S34] |

**Supplementary References**

[S1] X. G. Sun, W. Shen, H. Liu, P. X. Xi, M. Jaroniec et al., Corrosion-resistant NiFe anode towards kilowatt-scale alkaline seawater electrolysis. Nat. Commun. **15**, 10351 (2024). <https://doi.org/10.1038/s41467-024-54754-5>
[S2] M. F. Lagadec, A. Grimaud, Water electrolysers with closed and open electrochemical systems. Nat. Mater. **19**, 1140–1150 (2020). <https://doi.org/10.1038/s41563-020-0788-3>
[S3] Y. Zuo, S. Bellani, M. Ferri, G. Saleh, D. M. Shinde et al., High-performance alkaline water electrolyzers based on Ru-perturbed Cu nanoplatelets cathode. Nat. Commun. **14**, 4680 (2023). <https://doi.org/10.1038/s41467-023-40319-5>
[S4] X. Luo, H. Y. Zhao, X. Tan, S. Lin, Y. S. Yu et al., Fe-S dually modulated adsorbate evolution and lattice oxygen compatible mechanism for water oxidation. Nat. Commun. **15**, 8293 (2024). <https://doi.org/10.1038/s41467-024-52682-y>
[S5] Y. Z. Chen, Q. H. Li, Y. X. Lin, J. Liu, J. Pan et al., Boosting oxygen evolution reaction by FeNi hydroxide-organic framework electrocatalyst toward alkaline water electrolyzer. Nat. Commun. **15**, 7278 (2024). <https://doi.org/10.1038/s41467-024-51521-4>
[S6] P. C. Ye, K. Q. Fang, H. Y. Wang, Y. H. Wang, H. Huang et al., Lattice oxygen activation and local electric field enhancement by co-doping Fe and F in CoO nanoneedle arrays for industrial electrocatalytic water oxidation. Nat. Commun. **15**, 1012 (2024). <https://doi.org/10.1038/s41467-024-45320-0>
[S7] L. Yu, Q. Zhu, S. W. Song, B. McElhenny, D. Z. Wang et al., Non-noble metal-nitride based electrocatalysts for high-performance alkaline seawater electrolysis. Nat. Commun. **10**, 5106 (2019). <https://doi.org/10.1038/s41467-019-13092-7>
[S8] Y. J. Mei, J. L. Chen, Q. Wang, Y. Q. Guo, H. W. Liu et al., MoZn-based high entropy alloy catalysts enabled dual activation and stabilization in alkaline oxygen evolution. Sci. Adv. **10**(47), eadq6758 (2024). <https://doi.org/10.1126/sciadv.adq6758>
[S9] D. Y. Li, R. Xiang, F. Yu, J. S. Zeng, Y. Zhang et al., In situ regulating cobalt/iron oxide-oxyhydroxide exchange by dynamic iron incorporation for robust oxygen evolution at large current density. Adv. Mater. **36**(5), 2305685 (2024). <https://doi.org/10.1002/adma.202305685>
[S10] J. X. Zhang, Y. H. Tu, X. M. Xu, J. Ke, L. H. Zhang et al., A high-entropy antiperovskite nitride enables efficient anion exchange membrane water electrolysis. Adv. Mater. **37**(38), 2509042 (2025). <https://doi.org/10.1002/adma.202509042>
[S11] J. Hu, T. Q. Guo, X. Y. Zhong, J. Li, Y. J. Mei et al., In situ reconstruction of high entropy heterostructure catalysts for stable oxygen evolution electrocatalysis under industrial conditions. Adv. Mater. **36**(14), 2310918 (2024). <https://doi.org/10.1002/adma.202310918>
[S12] A. Li, X. X. Tang, R. J. Cao, D. C. Song, F. Z. Wang et al., Directed surface reconstruction of Fe modified Co_2_VO_4_ spinel oxides for water oxidation catalysts experiencing self-terminating surface deterioration. Adv. Mater. **36**(31), 2401818 (2024). <https://doi.org/10.1002/adma.202401818>
[S13] S. Jo, J. I. Jeon, K. H. Shin, L. T. Zhang, K. B. Lee et al., Stabilization of lattice oxygen evolution reactions in oxophilic Ce-mediated Bi/BiCeO_1.8_H electrocatalysts for efficient anion exchange membrane water electrolyzers. Adv. Mater. **36**(27), 2314211 (2024). <https://doi.org/10.1002/adma.202314211>
[S14] F. R. Qian, L. S. Peng, D. F. Cao, W. Jiang, C. S. Hu et al., Asymmetric active sites originate from high-entropy metal selenides by joule heating to boost electrocatalytic water oxidation. Joule **8**(8), 2342–2356 (2024). <https://doi.org/10.1016/j.joule.2024.06.004>
[S15] Z. B. Li, R. Y. Wu, D. B. Duan, X. J. Liu, R. Li et al., Empowering multicomponent alloys with unique nanostructure for exceptional oxygen evolution performance through self-replenishment. Joule **8**(10), 1–18 (2024). <https://doi.org/10.1016/j.joule.2024.06.023>
[S16] Y. J. Zhang, W. Y. Zhang, X. W. Zhang, X. Wang, J. J. Wang et al., Activating lattice oxygen based on energy band engineering in oxides for industrial water/saline oxidation. Energy Environ. Sci. **17**(10), 3347–3357 (2024). <https://doi.org/10.1039/d4ee00214h>
[S17] Q. P. Huang, G. J. Xia, B. Huang, D. L. Xie, J. Wang et al., Activating lattice oxygen by a defect-engineered Fe_2_O_3_-CeO_2_ nano-heterojunction for efficient electrochemical water oxidation. Energy Environ. Sci. **17**(14), 5260–5272 (2024). <https://doi.org/10.1039/d4ee01588f>
[S18] H, S. Cao, X. Wen, X. Z. Luo, L. L. Ma, Z. A. Xu et al., Dual-site bridging mechanism for bimetallic electrochemical oxygen evolution. Angew. Chem. Int. Ed. **63**(46), e202411683 (2024). <https://doi.org/10.1002/anie.202411683>
[S19] B. H. Yao, Y. T. Chen, Y. Y. Yan, Y. Yang, H. H. Xing et al., Iron-induced localized oxide path mechanism enables efficient stable water oxidation. Angew. Chem. Int. Ed. **64**(4), e202416141 (2025). <https://doi.org/10.1002/anie.202416141>
[S20] M. T. Huo, H. Sun, Z. Jin, W. Liu, Y. Liang et al., Tailoring octahedron-te hedron synergism in spinel catalysts for acidic water electrolysis. J. Am. Chem. Soc. **147**(12), 10678–106895 (2025). <https://doi.org/10.1021/jacs.5c00665>
[S21] Z. H. Yin, Y. Huang, K. P. Song, T. T. Li, J. Y. Cui et al., Ir single atoms boost metal-oxygen covalency on selenide-derived NiOOH for direct intramolecular oxygen coupling. J. Am. Chem. Soc. **146**(10), 6846–6855 (2024). <https://doi.org/10.1021/jacs.3c13746>
[S22] Y. Huang, Z. Wang, H. Xiao, Q. Liu, X. Wang, Activating and stabilizing lattice oxygen via self-adaptive Zn-NiOOH sub-nanowires for oxygen evolution reaction. J. Am. Chem. Soc. **146**(42), 29006–29016 (2024). <https://doi.org/10.1021/jacs.4c09931>
[S23] A. Kumar, M. Gil-Sepulcre, J. P. Fandré, O. Rüdiger, M. G. Kim et al., Regulating local coordination sphere of Ir single atoms at the atomic interface for efficient oxygen evolution reaction. J. Am. Chem. Soc. **146**(48), 48, 32953–32964 (2024). <https://doi.org/10.1021/jacs.4c08847>
[S24] L. Y. Xiao, C. Q. Cheng, T. T. Yang, J. T. Zhang, Y. J. Han et al., A “two-pronged” strategy to boost hydrogen evolution kinetics on NiFe-based (oxy)hydroxides via oxygen deficient Ni-Mo-Fe coordinate structures for ultra-stable ampere-level alkaline overall water splitting. Adv. Mater. **36**(44), 2411134 (2024). <https://doi.org/10.1002/adma.202411134>
[S25] X. Y. Lu, Z. Y. Ma, Y. N. Chang, S. S. Wang, X. X. Li et al., Mott-schottky construction boosted plasma thermal and electronic effects on the Ag/CoV-LDH nanohybrids for highly efficient water oxidation. Adv. Mater. **36**(29), 2313057 (2024). <https://doi.org/10.1002/adma.202313057>
[S26] Z. J. Liang, D. Shen, Y. Wei, F. F. Sun, Y. Xie et al., Modulating the electronic structure of cobalt vanadium bimetal catalysts for high-stable anion exchange membrane water electrolyzer. Adv. Mater. **36**(41), 2408634 (2024). <https://doi.org/10.1002/adma.202408634>
[S27] C. Q. Li, B. Kim, Z. P. Li, R. Thapa, Y. F. Zhang et al., Direct electroplating ruthenium precursor on the surface oxidation nickel foam for efficient and stable bifunctional alkaline water electrolysis. Adv. Mater. **36**(31), 2403151 (2024). <https://doi.org/10.1002/adma.202403151>
[S28] N. Wang, D. Chen, S. L. Ning, J. Y. Lao, J. C. Xu et al., Fe cluster modified Co_9_S_8_ heterojunction: highly efficient photoelectrocatalyst for overall water splitting and flexible zinc-air batteries. Adv. Mater. **36**(2), 2306138 (2024). <https://doi.org/10.1002/adma.202306138>
[S29] Y. Zhou, F. Li, B. Dong, Y. Chai, Double self-reinforced coordination modulation constructing stable Ni^4+^ for water oxidation. Energy Environ. Sci. **17**(4), 1468 (2024). <https://doi.org/10.1039/d3ee02627b>
[S30] M. M. Jiang, J. Xu, Y. J. Chen, L. Q. Wang, Q. Zhou et al., Interstitial doping in ultrafine nanocrystals for efficient and durable water splitting. Angew. Chem. Int. Ed. **64**(14), e202424195 (2025). <https://doi.org/10.1002/anie.202424195>
[S31] S. Z. Luo, C. C. Dai, Y. K. Ye, Q. Wu, J. R. Wang et al., Elevated water oxidation by cation leaching enable tunable surface reconstruction. Angew. Chem. Int. Ed. **63**(31), e202402184 (2024). <https://doi.org/10.1002/anie.202402184>
[S32] W. J. Zhang, L. Yang, Z. Li, G. Z. Nie, X. J. Cao et al., Regulating hydrogen/oxygen species adsorption via built-in electric filed-driven electron transfer behavior at the heterointerface for efficient water splitting. Angew. Chem. Int. Ed. **63**(16), e202400888 (2024). <https://doi.org/10.1002/anie.202400888>
[S33] Y. W. Liu, L. R. Wang, R. Hübner, J. Kresse, X. M. Zhang et al., Cobalt-based Co_3_Mo_3_N/Co_4_N/Co metallic heterostructure as a highly active electrocatalyst for alkaline overall water splitting. Angew. Chem. Int. Ed. **63**(14), e202319239 (2024). <https://doi.org/10.1002/anie.202319239>
[S34] J. L. Gu, L. X. Li, Q. Yang, F. B. Tian, W. Zhao et al., Twinning engineering of platinum/iridium nanonets as turing-type catalysts for efficient water splitting. J. Am. Chem. Soc. **146**(8), 5355–5365 (2024). <https://doi.org/10.1021/jacs.3c12419>
